# Chemical Profile, Antioxidant and Antibacterial Activities, Mechanisms of Action of the Leaf Extract of *Aloe arborescens* Mill

**DOI:** 10.3390/plants12040869

**Published:** 2023-02-15

**Authors:** Tsolanku Sidney Maliehe, Mduduzi Innocent Nqotheni, Jabulani Siyabonga Shandu, Tlou Nelson Selepe, Peter Masoko, Ofentse Jacob Pooe

**Affiliations:** 1 Department of Biochemistry and Microbiology, Faculty of Science and Agriculture, University of Zululand, Private Bag X1001, Empangeni 3886, South Africa; 2 Department of Water and Sanitation, University of Limpopo, Private Bag X1106, Polokwane 0727, South Africa; 3 Department of Biochemistry, Microbiology and Biotechnology, University of Limpopo, Private Bag X1106, Polokwane 0727, South Africa; 4 School of Life Science, Discipline of Biochemistry, University of KwaZulu-Natal, Durban 4000, South Africa

**Keywords:** *Aloe arborescens* Mill, phytochemicals, antioxidants, antibacterial effect, antibacterial mechanisms

## Abstract

*Aloe arborescens* Mill’s extracts have been explored for antibacterial and antioxidant efficacies. However, there is limited information on its chemical composition and mechanism of action. The purpose of this study was to assess the chemical composition, antibacterial and antioxidant activities and mechanism of the whole leaf extract of *A. arborescens* Mill. The phytochemical profile was analysed with gas chromatography mass spectrometry (GC-MS). The antioxidant and antibacterial activities were screened using 1,1diphenyl2picrylhydrazyl (DPPH), 2,2′-azino-bis(3-ethylbenzthiazoline-6-sulfonic acid) (ABTS) and micro-dilution assays, respectively. The effects of the extract on the bacterial respiratory chain dehydrogenase, membrane integrity and permeability were analysed using iodonitrotetrazolium chloride, 260 absorbing materials and relative electrical conductivity assays. GC-MS spectrum revealed 26 compounds with N,N’-trimethyleneurea (10.56%), xanthine (8.57%) and 4-hexyl-1-(7-ethoxycarbonylheptyl)bicyclo[4.4.0]deca-2,5,7-triene (7.10%), being the major components. The extract also exhibited antioxidant activity with median concentration (IC_50_) values of 0.65 mg/mL on DPPH and 0.052 mg/mL on ABTS. The extract exhibited minimum inhibitory concentration (MIC) values ranging from 0.07 to 1.13 mg/mL. The extract inhibited the bacterial growth by destructing the activity of the respiratory chain dehydrogenase, membrane integrity and permeability. Therefore, the leaf extract has the potential to serve as a source of antibacterial and antioxidant compounds.

## 1. Introduction

Free radicals are highly unstable chemical species with an unpaired electron in their outermost shell [1]. They are essential in many biological processes; at low to moderate amounts, free radicals regulate and maintain homeostasis [2]. However, an aberrant bioproduction of free radicals often results in the onset of oxidative stress, a condition which results in oxidative damage of cellular components (proteins, lipids, and nucleic acids) and disruption of metabolism [3]. Thus, more often, oxidative stress is implicated in the development of different pathologies such as cancer, diabetes and arthritis [4]. Antioxidant molecules, both of enzymatic or non-enzymatic origin, counteract the oxidation stress by maintaining the redox balance and preventing disturbances of the redox homeostasis. They achieve this by significantly inhibiting the over production of free radicals, scavenging and/or reducing the existing radicals [5]. Synthetic antioxidants such as butylated hydroxytoluene and butylated hydroxyanisole (BHA) are currently predominantly used because of their high stability, efficacy and availability [6]. However, they have been reported to be costly and to have undesirable side effects [1]. Therefore, health concerns and high costs of synthetic antioxidants have sparked an interest in natural antioxidants, particularly those of plant origin [7,8].

For centuries, antimicrobials have played an important role in human health as they effectively treat microbial infections. However, with their vast use, microorganisms develop different countermeasures to combat the impact of antimicrobials [9]. These have resulted in antimicrobials having a short life expectancy, with resistance developing within a year after their introduction and others developing resistance in less than 20 years [10]. The ever increasing prevalence of antimicrobial resistance is strongly linked to different factors such as over prescription, inappropriate doses, easy accessibility over the counter and of course the rise in new pathogens [11]. The magnitude of antimicrobial resistance is translated into high mortality rate and drastic economic crisis. Apparently, about 700,000 patients die annually due to antimicrobial resistant infections, and these infections are also projected to cause 10 million deaths per year by the year 2050 [12]. Moreover, over 28.3 million people are estimated to live in extreme poverty in 2050 due to the impact of antimicrobial resistance [13]. Therefore, there is a need for novel antimicrobials to overcome the crisis of resistance.

The connection between man and his search for medicines of plant origin dates from the far past [14]. It is estimated that 60% of the world’s population depends directly on plant-based medicine for their health care [15]. Plant-based medicines are often prescribed, especially in developing countries, even if their chemical constituents are not completely known. Moreover, in most cases, the mechanisms of action of these phyto-chemicals are undefined [16]. However, the knowledge of the chemical composition and mode of action of plant-based extracts is critical in solving the antimicrobial resistance crisis and can result in the use of appropriate dosages during treatment, which can translate into low costs and high biosafety levels.

*Aloe arborescens* is one of the widely used medicinal plants belonging to the genus *Aloe* and *Asphodeceae* family [17]. It is widely distributed in the south-eastern half of Africa, especially in South Africa, Malawi, Lesotho, Mozambique and Zimbabwe [18]. Traditionally, the aloe is used as a remedy for asthma, stomach-ache, tuberculosis, HIV/AIDS, burning injuries and abrasions [19,20]. The leaves of *A. arborescens* are divided into two main parts, namely, the outer part called rind and the inner parenchyma [21]. Moreover, the leaves have been the most studied part of this aloe, and their extracts have revealed wound healing, antibacterial, anti-ulcer, anti-inflammatory, antioxidant, anti-cancerous and alopecia-relieving properties [22]. Kumar et al. [23] have reported the presence of glycosides, anthraquinones, flavonoids, chromones, anthrones, coumarins and pyrones in its leaves, which are well recognised for diverse pharmacological activities [21]. Nevertheless, according to the literature, most of the studies on the medicinal efficacy of aloes and their phytochemical composition have focused on *Aloe vera*, leaving other species such as *A. arborescens* unexplored [24]. Furthermore, although the leaves of *A. arborescens* Mill have been the most pharmacologically studied part, especially their antibacterial activity, according to our knowledge, no study has recorded their mechanisms of antibacterial action.

This study aimed to determine the chemical profile, in vitro antioxidant and antibacterial activities of the whole leaf extract from *A. arborescens.* Moreover, the mechanisms of antibacterial action of the extract were also evaluated.

## 2. Results

### 2.1. GC-MS Profile of the Whole Leaf Extract of A. arborescens Mill

GC-MS spectrum revealed about 26 compounds. N,N’-trimethyleneurea was the main constituent (10.56%), followed by xanthine (8.57%), 4-hexyl-1-(7-ethoxycarbonylheptyl)bicyclo[4.4.0]deca-2,5,7-triene (7.10%), and indole (5.03%). Other compounds, in a lesser quantity, were decanoic acid, 10-bromo-, trimethylsilyl ester (1.63%), cyclohexanone, 2,6-diethyl (1.91%), cyclopropanebutanoic acid, 2-[[2-[[2-[(2-pentylcyclopropyl)methyl]cyclopropyl]methyl]cyclopropyl]methyl]-, methyl ester (1.94%) and 1,7-Dioxa-10-thia-4,13-diazacyclopentadeca-5,9,12-trione (1.95%) (Table 1 and Supplementary Materials Figure S1).

### 2.2. Antioxidant Activity of the Whole Leaf Extract

The antioxidant efficacy of the whole leaf extract was assessed in vitro, and the results are displayed in Table 2. The IC_50_ value of the leaf extract was higher than that of ascorbic acid and BHA in all assays, as it was determined to be 0.065 against DPPH and 0.052 mg/mL against ABTS.

### 2.3. Antibacterial Activity of the Whole Leaf Extract

The antibacterial potency of the whole leaf extract of *A. arborescens* Mill was evaluated against the 4 selected strains, and the results are shown in Table 3. High level of sensitivity was observed against Gram-positive bacteria in comparison to the Gram-negative bacteria, with *S. aureus* being the most susceptible strain with an MIC value of 0.07 mg/mL. *E. coli* was the most resistant with an MIC value of 1.13 mg/mL. Moreover, the extract demonstrated bactericidal effect against all tested strains except for *E. coli.* The lowest bactericidal concentration of 1.25 mg/mL was effective against the Gram-positive bacteria (*S. aureus* and *E. faecalis*).

### 2.4. Effect of the Extract on the Bacterial Respiratory Chain Dehydrogenase

**Figure 1:**
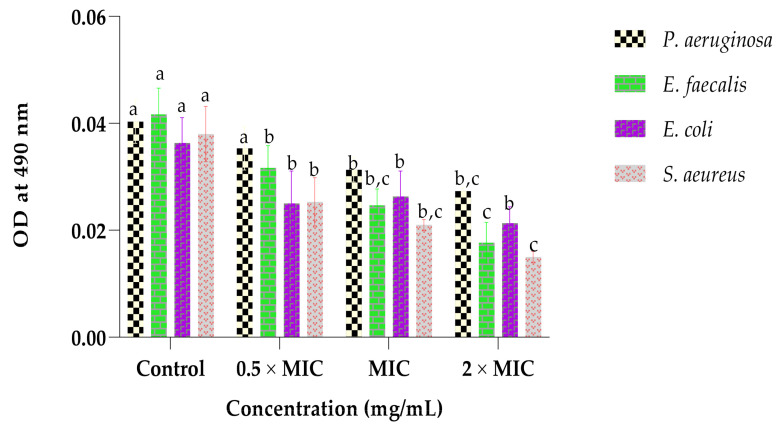
Effect of the whole leaf extract on the respiratory chain dehydrogenase activity of *P. aeruginosa*, *E. faecalis*, *E. coli* and *S. aureus*. The different superscripts (a, b and c) illustrate the statistically significant (*p* < 0.05) per bacterium; similar letters indicate no statistical difference (*p* > 0.05).

Figure 1 displays the inhibitory effect of the whole leaf extract on the bacterial respiratory chain dehydrogenase activity. The extract affected the respiratory chain dehydrogenase activity in a dose-dependent manner against all test bacterial strains. The highest and lowest effects were observed when 2 × MIC and 0.5 × MIC concentrations were used, respectively. Moreover, the effect of the extract (2 × MIC) was maximum on S. aureus, revealing the OD of 0.015, and was lowest on *P. aeruginosa*, with the OD of 0.027. It was also noted that the respiratory chain dehydrogenase activity was greatly interrupted by the extract on the Gram-positive bacteria (*S. aureus* and *E. faecalis*) than on Gram-negative strains (*P. aeruginosa* and *E. coli*). The control showed maximum OD readings in all experiments.

### 2.5. Effect of the Leaf Extract on the Bacterial Cell Integrity

**Figure 2:**
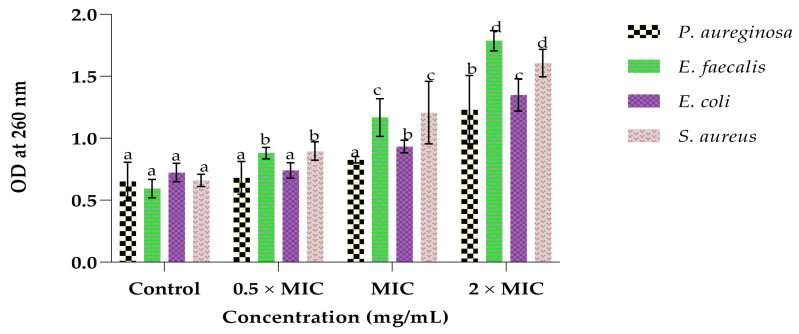
Effect of the whole leaf extract on the release of 260 nm absorbing materials by *P. aeruginosa*, *E. faecalis*, *E. coli* and *S. aureus*. The different superscripts (a, b, c and d) represent the statistical difference (*p* < 0.05) per bacterium; similar letters indicate no statistical difference (*p* > 0.05).

The effect of the leaf extract on the integrity of the the bacterial cell membranes of the tested bacteria was investigated and the results are illustrated in Figure 2. There was an increase in the OD with the increase in the concentration of the extract. The highest OD was shown at the highest concentration for all bacterial suspensions. Moreover, the OD values of the Gram-positive bacteria were found to be higher than those of the Gram-negative bacteria in all treatments. *E. faecalis* had the highest OD reading of 179, whereas *P. aureginosa* had the least value of 1.23 at the highest concentration (2 × MIC). Nevertheless, the OD readings of the treated bacterial suspensions were greater than those of the controls.

### 2.6. Effect of the Extract on the Bacterial Cell Membrane Permeability

**Figure 3:**
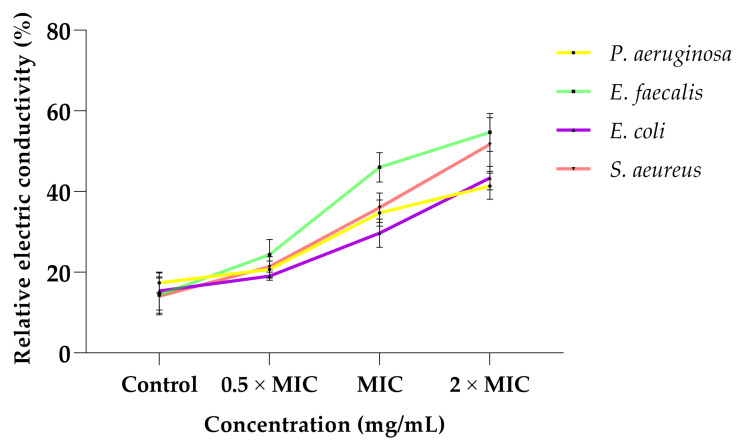
The effect of the whole leaf extract on the cell membranes of *P. aeruginosa*, *E. faecalis*, *E. coli* and *S. aureus*.

Figure 3 demonstrates the effect of the whole leaf extract on the membrane permeability of the four selected bacteria. The conductivity of the treated bacteria illustrated a dependent relationship, and there was an increase in conductivity observed when the concentration was increased. The highest concentration (2 × MIC) had the maximum conductivities against all bacterial strains. *E. faecalis* revealed the highest conductivity (54.7%) after being treated with 2 × MIC, while *P. aeruginosa* had the least relative electric conductivity (41.3%). The control revealed the lowest conductivity.

## 3. Discussion

The high cost and side effects of aliphatic drugs, the sluggish pace of antimicrobial drug development and the discovery of new antimicrobials necessitate the search for novel antimicrobial agents [25]. Aloe species are well documented for their pharmaceutical importance as they have diverse bioactivities, which include antimicrobial and antioxidant properties [26,27].

The GC-MS analysis revealed the presence of phytochemicals known to induce antibacterial and antioxidant activities. For instant, the main compounds, xanthine, morpholine, octanoic and indole, possesses antimicrobial and antioxidant properties [28,29,30,31]. According to the literature, there are limited studies that have published the phytoconstituents of *A. arborescens.* Thus, most of the phytoconstituents detected in this study, such as xanthine, octanoic, indole and decanoic acid derivatives, have been previously identified from other Aloe species such as *Aloe ferox* and *A. vera* but not recorded from *A. arborescens* [32,33,34]. Therefore, the presence of these compounds implies that *A. arborescens* has potential pharmacological activities and can be considered in medical prophylactic and therapeutic schemes.

The leaf extract illustrated good ABTS scavenging activity and highly active DPPH scavenging action. According to Nxumalo et al. [35], the extract is regarded to be highly active when its IC_50_ value is <0.05 mg/mL, medium when IC_50_ value is in the range of 0.1–0.15 mg/mL and weak when IC_50_ value is in the range of 0.151–0.2 mg/mL. This implied that the extract has potential to maintain a normal redox state in biological systems and ability to reduce the risk of various diseases development emanating due to oxidative damage. Our findings affirmed those obtained by Cardarelli et al. [36], whereby the leaf exudants from *A. arborescens* revealed profound DPPH scavenging activity. Moreover, the study by Andrea et al. [37] revealed the whole leaf extract from *A. arborescens* to have significantly better DPPH scavenging activity in comparison to other aloes such as *Aloe aculeate*, *Aloe Africana*, *Aloe barbadensis*, *Aloe ferox*, *Aloe marlothii* and *Aloe spectabilis.* Furthermore, the study by Pawłowicz et al. [38] also affirmed the antioxidant potency of the leaf extracts of *A. arborescens* by their ability to demonstrate ABTS savaging properties. The profound antioxidant activity can be due to the synergistic effect of the detected phytocompounds within the extract [39].

The antibacterial efficacy of the whole leaf extract from *A. arborescens* Mill was evaluated, and the extract demonstrated a broad spectrum antibacterial potency. The extract revealed MIC values less than 1 mg/mL against the selected strains, indicative of its noteworthiness [40]. Furthermore, the extract did not only demonstrate the bacteriostatic effect but also the bactericidal effect on the tested bacteria (except on *E. coli*). Moreover, the sensitivity of the Gram-positive bacteria to the extract in comparison to the Gram-negative bacteria was perceived to be due to the differences in their cell membranes, as the Gram-negative bacteria possess phospholipid membranes which comprise the structural lipopolysaccharide components, which enhance their resistance to most antibacterial agents [41]. The results were in agreement with those of Bisi-Johnson et al. [42], whereby the leaf extract of A. arborescence revealed a noteworthy broad-spectrum antibacterial activity.

The disturbance of the respiratory system, membrane integrity and permeability of bacteria are regarded as targets for antibacterial agents. The bacterial respiratory chain dehydrogenase is responsible for the generation of the electrochemical gradient, which, in turn contributes to the energy production. Thus, the disturbance of the respiratory chain dehydrogenase can result in insufficient energy production and supply, consequently leading to the bacterial inhibition and/or death [43]. In this study, the extract showed destructive properties against the respiratory chain dehydrogenase, implying that the bacteria’s energy production pathway was affected, consequently leading to the inhibition and/or killing of the selected bacterial strains [44]. The results are in agreement with the findings by Gomaa, [45], whereby the antimicrobial compounds were able to exert their efficacies by tempering with the activity of bacterial respiratory chain dehydrogenase.

The bacterial cell membrane is essential for blocking extracellular materials from entering the cell, and it also maintains the cell stability of the intracellular environment. When the bacterial cell membrane is destroyed by antimicrobials, biomolecules such as nucleic acids (DNA and RNA) leak out of the cell due to the high permeability. DNA and RNA have maximum absorption peaks at 260 nm [46]. In this study, the leaf extract was efficacious in inhibiting or killing the bacteria by damaging their cell membranes, resulting in the leakage of the 260 nm absorbing materials, such as DNA and RNA, which are essential for bacterial growth [47]. These results were in agreement with those obtained by Tang et al. [48], whereby the extract inhibited the tested bacteria by destructing their cell membranes.

The observed increase in the relative electrical conductivity of the treated bacteria in comparison to the untreated meant that the extract exhibited antibacterial effect by affecting the membrane structures of the selected bacteria. The increase in the electric conductivity of bacteria suspensions implied that the permeability of the bacterial cell membranes were increased, resulting in the leakage of intracellular ingredients (e.g., electrolytes) and increase in conductivity. Electrolytes are charged molecules such as sodium chloride and potassium chloride, and they are essential for bacterial metabolism and growth [49]. Thus, their leakage can lead to bacterial inhibition or death. Moreover, the differences in the relative electric conductivity between Gram-positive bacteria and Gram-negative bacteria suggest that the extract had a better effect on permeating membranes of the Gram-positive bacteria than those of the Gram-negative bacteria. Similar trends were recorded by Li et al. [50], where the plant essential oil exerted its broad antibacterial spectrum by interfering with the cell membranes of the tested strains. Therefore, it was concluded that the extract has potential applicability as a source of antibacterial agent. Generally, the profound antibacterial and antioxidant activities can be postulated due to the synergistic effect of the multiple phytocompounds within the extract [51].

## 4. Materials and Methods

### 4.1. Chemicals and Media

The chemicals and culture media used in this study were of analytic grade and were procured from Sigma-Aldrich and Merck (Pty) Ltd., Johannesburg, South Africa. The water was distilled and autoclaved (121 °C for 15 min) at the University of Zululand, KwaZulu Natal, South Africa.

### 4.2. Bacterial Strains

The American Type Culture Collection (ATTC) bacterial strains (*Staphylococcus aureus* (ATCC 25925), *Enterococcus faecalis* (ATTC 29212)*, Pseudomonas aeruginosa* (ATCC 27853) and *Escherichia coli* (ATCC 25922)) were obtained from the Microbiology Culture Bank at the Department of Biochemistry and Microbiology, University of Zululand.

### 4.3. Plant Selection and Sampling

*A. arboscenes* Mill was selected based on its history of use in South African traditional medicine. Fresh leaves of the aloe were obtained in March 2021 at the University of Zululand, KwaDlangezwa campus, South Africa (latitude 28.753° S, longitude 31.894° E, altitude 117 m). Its voucher specimen number (MN01) was deposited in the University of Zululand Herbarium [ZULU]. Ethical approval to collect the plant was acquired from the research ethical committee at the University of Zululand (UZREC 171110-030 PGM 2021/56). The whole leaves of the aloe were washed with tap water to remove soil and debris, air-dried in the fume hood and ground to powder.

### 4.4. Extraction of the Phytochemicals

Hundred millilitres of a mixture of ethanol (70%) and methanol (80%) at a ratio of 1:10 was added to extract the phytochemicals from the ground aloe powder (10 mg). After 3 days of extraction, the extract was filtered using Whatman No.1 filter paper and subsequently concentrated by evaporating the solvents under fume hood. The extract was re-constituted in acetone and made to the final concentration of 10 mg/mL [52].

### 4.5. Analysis of Volatile Phytochemicals of the Extract

The analysis of the compounds within the whole leaf extract was performed using gas chromatography–mass spectrophotometer (THERMO Gas Chromatography TRACE ULTRA VER: 5.0.). In short, the helium gas flow rate was set to 1 mL per minute, with the split ratio of 1:50. The injector temperature was programmed to 250 °C with the detector temperature set to 280 °C. The temperature of the column was adjusted to 40 °C for a minute, and it was programmed to increase to 120 °C thereafter. About 2 µL of the extract was injected for analysis, and the mass spectra was programmed in the scan mode was 70 eV [53].

### 4.6. Antioxidant Activity of the Extract

#### 4.6.1. DPPH Radical Scavenging Activity of the Extract

The in vitro antioxidant activity of the whole leaf extract was determined by evaluating the DPPH free radical scavenging activity using ultraviolet-visible (UV-Vis) spectrophotometry at 517 nm according to Brand-Williams [54]. The DPPH (0.02 mg/mL) was mixed (1:1 *v/v*) with different concentrations of the extract. Each mixture was made to stand for 30 min in darkness at room temperature (25 °C), and the absorbance was read at 517 nm using a microplate reader (MODEL). The extract without DPPH served as blank, while ascorbic acid (AA) and butylated hydroxyl anisole (BHA) were used as the positive controls. The percent inhibition of DPPH radical was calculated using the formula:

%DPPH scavenging activity = [A_z_ − A_w_/A_z_] × 100,

whereby A_z_ and A_w_ represent the absorbance recorded at 517 nm for the control and the test, respectively. The median inhibitory concentrations (IC_50_) of the leaf extract and the controls were calculated using the linear regression analysis.

#### 4.6.2. ABTS Radical Scavenging Activity of the Extract

The standard ABTS technique was employed in vitro to assess the scavenging activity of the extract using UV-Vis spectrophotometry at 734 nm. Briefly, ABTS solution (0.003 g/mL) was mixed with different concentrations of the extract (1:1 *v/v*). The mixtures were made to stand for 15 min at 25 °C, and the absorbance was read at 734 nm using a microplate reader. The extract without ABTS solution served as a blank; ascorbic acid (AA) and butylated hydroxyl anisole (BHA) were used as the positive controls. The percent inhibition of ABTS radical was obtained using the formula:

%ABTS scavenging activity = [A_z_ − A_w_/A_z_] × 100,

where A_z_ and A_w_ equal the absorbance recorded at 734 nm of the control and the test, respectively. The median inhibitory concentration (IC_50_) of the extract and controls against ABTS was calculated graphically. The IC_50_ of the whole leaf extract and the controls were calculated using the linear regression analysis [55].

### 4.7. Determination of Antibacterial Activity

#### 4.7.1. MIC of the Leaf Extract

The whole leaf extract was subjected to antibacterial analysis by evaluating its MIC using a rapid Mueller Hinton broth micro-dilution method with iodonitrotetrazodium violet (INT) solution (0.2 mg/mL) as an indicator [56]. Before the evaluation of MIC, the test bacteria, at exponential phase, were adjusted to 1 × 10^8^ colony forming units per millilitre (CFU/mL). Acetone served as the negative control, while ciprofloxacin as the positive control.

#### 4.7.2. MBC of the Extract

The MBC was assessed by using 20 µL of bacterial suspensions from the wells that demonstrated no growth during the evaluation of MICs. The suspensions were pipetted into 50 µL of NB in a sterile 96-well plate. The plates were incubated at 37 °C, overnight. Thereafter, 40 µL of INT was pipetted in each well and the plates were re-incubated at 37 °C for 30 min. The lowest concentration that displayed no bacterial growth was identified as the MBC of the extract [57].

### 4.8. Determination of the Antibacterial Mechanisms of Action of the Extract

#### 4.8.1. Effect of the Extract on the Bacterial Respiratory Chain Dehydrogenase

The effect of the extract on the bacterial respiratory chain dehydrogenase activity of the test bacteria was evaluated using the iodonitrotetrazolium chloride (INT) technique. The bacteria were cultured on NB, incubated overnight at 37 °C and adjusted to 1 × 10^8^ CFU/mL. Thereafter, 1 mL of the bacterial suspensions was added into the sterile test tube, followed by addition of 2 mL of 0.05 mol/L Tris-HCl buffer (pH = 8.6), 2 mL of 0.1 mol/L glucose solution and 2 mL of 1 mg/mL triphenyl formazan solution. After mixing, the extract (0.5 × MIC, MIC and 2 × MIC) was pipetted and incubated at 37 °C for 6 h. Thereafter, 2 drops of a concentrated sulphuric acid (H_2_SO_4_) were pipetted into each test tube to stop the reaction, and 5 mL of n-butyl ethanol was added to extract the products. The upper organic phase was centrifuged at 5000 rpm for 15 min, its optical density at 490 nm was measured, and n-butyl ethanol was used to blank. The cells that were boiled for 30 min to inactivate the respiratory chain dehydrogenase served as the negative control, while the positive control was the cells that were not boiled [58].

#### 4.8.2. Effect of the Extract on the Integrity of the Bacterial Cell Membranes

The cell integrity of the bacterial strains was examined by determining the release of cell constituents into supernatant. Bacteria were cultured at 37 °C for 24 h in 10 mL nutrient broth and then centrifuged at 8000 rpm for 15 min, washed twice, and re-suspended in sterile saline solution (0.85% NaCl). A volume of 5 mL of the bacterial suspensions was incubated at 37 °C under agitation (150 rpm) for 4 h in the presence of the extract (0.5 × MIC, MIC and 2 × MIC). The control was cells without extract. Thereafter, the mixtures were centrifuged at 8000 rpm for 10 min. To determine the concentration of the constituents released, the absorption of the supernatants were at 260 nm [59].

#### 4.8.3. Effect of the Extract on the Outer Bacterial Cell Membrane Permeability

Bacterial membrane permeability was determined and expressed as the relative electric conductivity according to the method by Hao et al. [49]. Bacterial cells were cultivated at 37 °C to mid-exponential stage and collected by centrifugation (8000 rpm for 15 min). Cells were washed twice in 5% glucose solution, and their conductivities were adjusted until they were equal or near the conductivity value of the washing buffer (5% glucose). The extract at different concentrations (0.5 × MIC, MIC and 2 × MIC) were diluted in 5% glucose, and their electric conductivities were measured and recorded as *A*_1_. The same concentrations were added into the isotonic bacterial suspensions (1 × 10^8^ CFU/mL) and incubated at 37 °C for 6 h. Thereafter, their conductivities were measured and recorded as A_2_. The conductivities of the bacteria in 5% glucose treated with boiling water for 5 min was used as the control and marked as A_0_. The cell membrane permeability was then calculated using the formula:

Relative electric conductivity (%) = (A_2_ − A_1_) A_0_ × 100.

### 4.9. Data Analysis

All experiments were performed in triplicate and data was expressed as mean ± standard deviation. The statistical analyses were performed by one-way analysis of variance and were considered to be significantly different at *p* < 0.05 and not different when *p* > 0.05.

## 5. Conclusions

The whole leaf extract of *A. arborescens* Mill revealed to have multiple phytochemicals known to possess pharmacological properties such as antioxidant and antibacterial activities. It was deduced that the extract has antioxidant activity, as illustrated by the DPPH and ABTS scavenging activities. Moreover, the whole leaf extract displayed noteworthy antibacterial activity, as evidenced by the low MIC and MBC values. The extract exerted its antibacterial effect by inhibiting the activity of the bacterial respiratory chain dehydrogenase. Moreover, the leakage of nucleic acids, due to the increase in the bacterial cell membrane permeability and the increase in the conductivity due to the leakage of electrolytes, implied that the extract of *A. arborescens* Mill inhibited the test bacteria by destructing their membrane structures and functions. For further studies, individual phytochemicals should be isolated and evaluated for their activity in vitro and in vivo.

## Figures and Tables

**Table 1:** Phytochemical compounds of the leaf extract as revealed by GC-MS.

Phytocompounds	Area %
Indole	5.03
Morpholine, 4-[3-(4-fluoro-3-nitrophenylsulfonyl)propyl]-	4.85
Octanoic acid	3.67
Acetate, (2-(3-hydroxy-3-methyl-2-oxotetrahydro-1H-1-pyrrolyl)ethyl] ester	3.72
N,N’-Trimethyleneurea	10.56
Thiazolo[3,2-a]pyridinium, 3-hydroxy-2-methyl-, acetate	2.70
1,1’-Bicyclohexyl, 2-(1-methylethyl)-, cis-	2.77
Xanthine	8.57
4-Hexyl-1-(7-methoxycarbonylheptyl)bicyclo[4.4.0]deca-2,5,7-triene	7.10
2,5-Di-O-acetyl-3,4,6-tri-O-methyl-D-gluconitrile	2.81
Hexa-t-butylcyclotrisilane	2.15
Methyl trans-9-(2-butylcyclopentyl)nonanoate	2.84
3-Propylglutaric acid, monomethyl ester	3.63
D-Galactitol, 3,6-anhydro-1,2,4,5-tetra-O-methyl	5.58
1,3-Cyclohexanediacetic acid, 2-oxo-, dimethyl ester	3.70
Pyrrolidin-2-one, 5-[3-ethylenedithio-1-pentyl]-	4.99
Decanoic acid, 10-bromo-, trimethylsilyl ester	1.63
Cyclohexanone, 2,6-diethyl-	1.91
2H-Furo[3,2-b]pyran-2-one, hexahydro-3,4(or 3,8)-dihydroxy-8(or 4)-methoxy-6,7,8-trimethyl-	2.78
Pyrrolo[1,2-a]pyrazine-1,4-dione, hexahydro-3-(2-methylpropyl)-	4.98
Pentanoic acid, 2-(methoxymethyl)-4-oxo-	3.02
4-Amino-furazan-3-carboxylic acid (2-acetylamino-ethyl)-amide	2.17
Cedran-diol, 8S,13-	2.40
3-(1,3-Dihydroxyisopropyl)-1,5,8,11-tetraoxacyclotridecane	2.61
Cyclopropanebutanoic acid, 2-[[2-[[2-[(2-pentylcyclopropyl)methyl]cyclopropyl]methyl]cyclopropyl]methyl]-, methyl ester	1.94
1,7-Dioxa-10-thia-4,13-diazacyclopentadeca-5,9,12-trione	1.95

**Table 2:** IC_50_ values of the leaf extract, ascorbic acid and BHA.

Assay	Leaf Extract (mg/mL)	Ascorbic Acid (mg/mL)	BHA (mg/mL)
DPPH	0.065 ± 1.64	0.022 ± 1.20	0.015 ± 0.57
ABTS	0.052 ± 2.54	0.019 ± 0.74	0.022 ± 2.71

**Table 3:** MIC and MBC of the whole leaf extract of *A. arborescens* Mill.

Bacteria	Leaf Extract		Ciprofloxacin	
	MIC (mg/mL)	MBC (mg/mL)	MIC (µg/mL)	MBC (µg/mL)
*S. aureus*	0.07	1.25	0.02	0.03
*E. faecalis*	0.14	1.25	0.02	0.06
*P. aeruginosa*	0.63	2.25	0.04	0.24
*E. coli*	1.13	>2.25	0.02	0.03

## Data Availability

The data generated or analysed are included in this article.

## Supplementary Materials

The following supporting information can be downloaded at: https://www.mdpi.com/article/10.3390/plants12040869/s1, Figure S1: GC-MS analysis of the whole leaf extract of *A. arborescens* Mill.
