# Supplementary material for: Chemical Profile, Antioxidant and Antibacterial Activities, Mechanisms of Action of the Leaf Extract of Aloe arborescens Mill
Source: Plants (Basel). 2023 Feb 15;12(4):869. doi: 10.3390/plants12040869 (PMC9968107; doi:10.3390/plants12040869)

Qualitative Analysis Report

Sample Information

Sample Type	: Unknown
Sample Name	: Sample 3 _2
Sample ID	:
Vial #	: 31
Injection Volume	: 3.00
Method File	: C:\GCMSData\general - split - extended.qgm
Tuning File	: C:\GCMSsolution\KAW\Normal Conc - 2022 01 26.qgt

Chromatogram Sample 3 _2

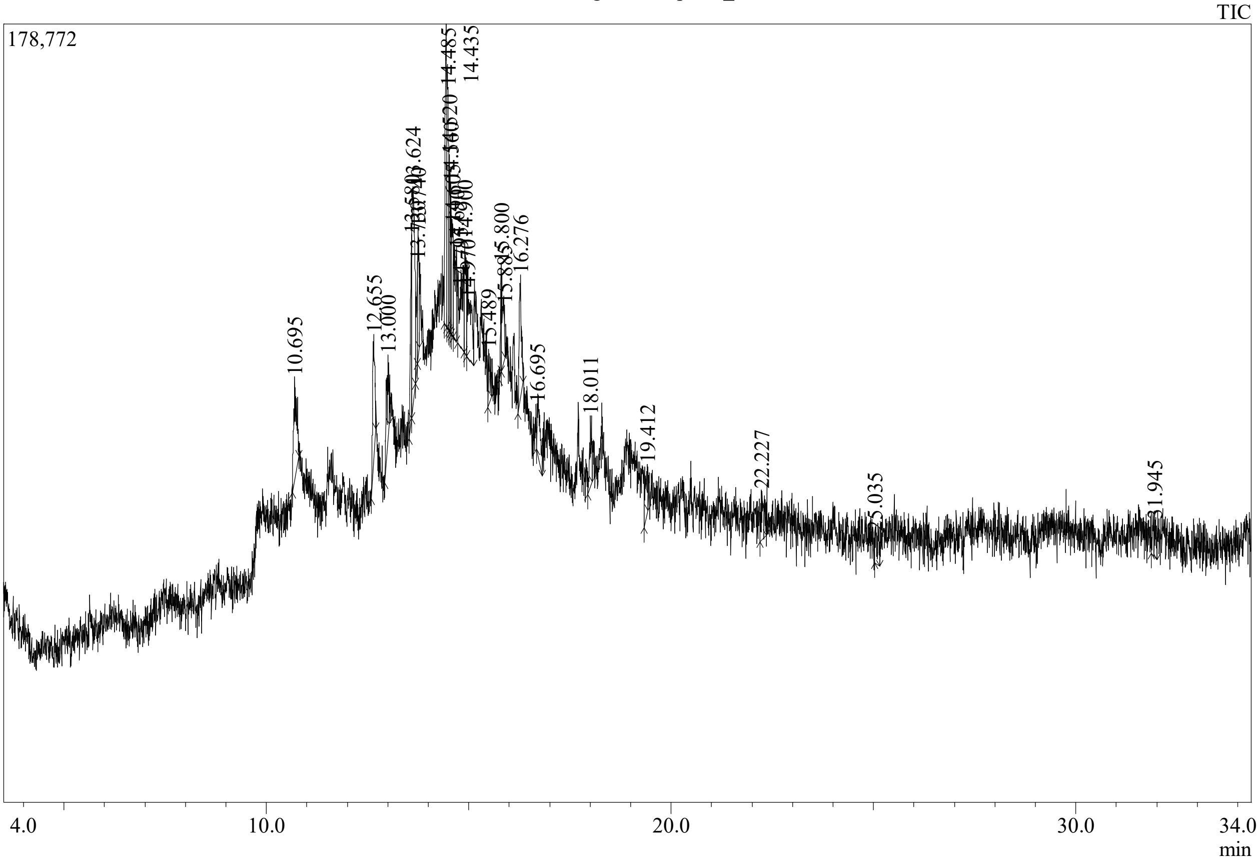

Peak Report TIC

Peak#	R.Time	Area	Area%	Height	Height%	A/H	Name
1	10.695	110288	5.03	23782	3.58	4.64	Indole
2	12.655	106269	4.85	27979	4.21	3.80	Morpholine, 4-[3-(4-fluoro-3-nitrophenylsulfor
3	13.000	80503	3.67	19883	2.99	4.05	Octanoic acid
4	13.580	81500	3.72	42606	6.42	1.91	Acetate, (2-(3-hydroxy-3-methyl-2-oxotetrahydı
5	13.624	231558	10.56	50032	7.54	4.63	N,N'-Trimethyleneurea
6	13.730	59127	2.70	23998	3.61	2.46	Thiazolo[3,2-a]pyridinium, 3-hydroxy-2-methyl
7	13.740	60816	2.77	30844	4.65	1.97	1,1'-Bicyclohexyl, 2-(1-methylethyl)-, cis-
8	14.435	187804	8.57	69357	10.45	2.71	Xanthine
9	14.485	155637	7.10	55520	8.36	2.80	4-Hexyl-1-(7-methoxycarbonylheptyl)bicyclo[4
10	14.520	61540	2.81	40536	6.11	1.52	2,5-Di-O-acetyl-3,4,6-tri-O-methyl-D-gluconitr
11	14.560	47073	2.15	34791	5.24	1.35	Hexa-t-butylcyclotrisilane
12	14.605	62210	2.84	25832	3.89	2.41	Methyl trans-9-(2-butylcyclopentyl)nonanoate
13	14.690	79516	3.63	21118	3.18	3.77	3-Propylglutaric acid, monomethyl ester
14	14.795	122268	5.58	13783	2.08	8.87	D-Galactitol, 3,6-anhydro-1,2,4,5-tetra-O-meth
15	14.900	81008	3.70	25976	3.91	3.12	1,3-Cyclohexanediacetic acid, 2-oxo-, dimethyl
16	14.970	109333	4.99	13265	2.00	8.24	Pyrrolidin-2-one, 5-[3-ethylenedithio-1-pentyl]
17	15.489	35824	1.63	12896	1.94	2.78	Decanoic acid, 10-bromo-, trimethylsilyl ester
18	15.800	41797	1.91	25396	3.82	1.65	Cyclohexanone, 2,6-diethyl-
19	15.885	60851	2.78	12387	1.87	4.91	2H-Furo[3,2-b]pyran-2-one, hexahydro-3,4(or [illegible]
20	16.276	108384	4.94	28228	4.25	3.84	Pyrrolo[1,2-a]pyrazine-1,4-dione, hexahydro-3-
21	16.695	66300	3.02	11092	1.67	5.98	Pentanoic acid, 2-(methoxymethyl)-4-oxo-
22	18.011	47530	2.17	16187	2.44	2.94	4-Amino-furazan-3-carboxylic acid (2-acetylam
23	19.412	52625	2.40	11288	1.70	4.66	Cedran-diol, 8S,13-
24	22.227	57274	2.61	11636	1.75	4.92	3-(1,3-Dihydroxyisopropyl)-1,5,8,11-tetraoxacy
25	25.035	42617	1.94	7094	1.07	6.01	Cyclopropanebutanoic acid, 2-[[2-[[2-[(2-penty
26	31.945	42689	1.95	8465	1.27	5.04	1,7-Dioxa-10-thia-4,13-diazacyclopentadeca-5,

Qualitative Analysis Report

Peak#	R.Time	Area	Area%	Height	Height%	A/H	Name
		2192341	100.00	663971	100.00		

Library

<< Target >>

Line#:1 R.Time:10.695(Scan#:1440) MassPeaks:382

RawMode:Averaged 10.690-10.700(1439-1441) BasePeak:117.10(5429)

BG Mode:Calc. from Peak Group 1 - Event 1 Scan

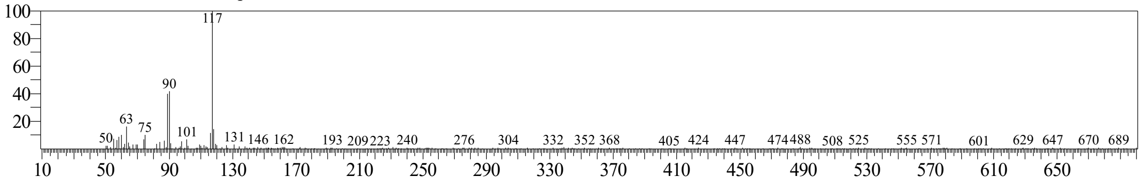

Hit#:1 Entry:4817 Library:NIST11.lib

SI:82 Formula:C8H7N CAS:120-72-9 MolWeight:117 RetIndex:1174

CompName:Indole $$ 1H-Indole $$ Ketole $$ 1-Azaindene $$ 1-Benzazole $$ 2,3-Benzopyrrole $$ Benzopyrrole $$ Indol $$

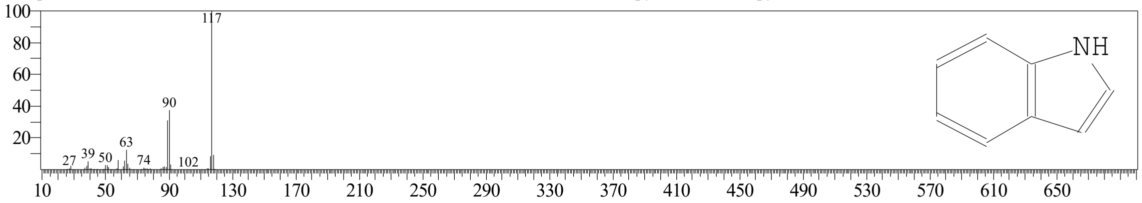

Hit#:2 Entry:4818 Library:NIST11.lib

SI:80 Formula:C8H7N CAS:274-40-8 MolWeight:117 RetIndex:991

CompName:Indolizine $$ Pyrrolo[1,2-a]pyridine $$ Indolizin $$ Pyrrocolin $$ Pyrrocoline $$

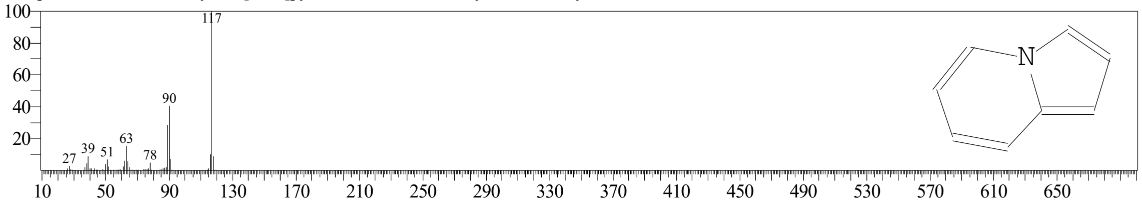

Hit#:3 Entry:4816 Library:NIST11.lib

SI:79 Formula:C8H7N CAS:270-91-7 MolWeight:117 RetIndex:1023

CompName:5H-1-Pyrindine $$ 5H-Cyclopenta[b]pyridine # $$

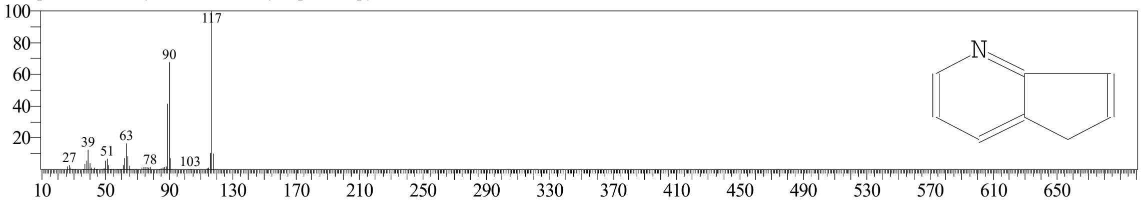

Hit#:4 Entry:4825 Library:NIST11.lib

SI:78 Formula:C8H7N CAS:620-22-4 MolWeight:117 RetIndex:1071

CompName:Benzonitrile, 3-methyl- $$ m-Tolunitrile $$ m-Cyanotoluene $$ m-Methylbenzonitrile $$ m-Toluenenitrile $$ m-Toluonitrile $$ 3-Methylbenzonitri

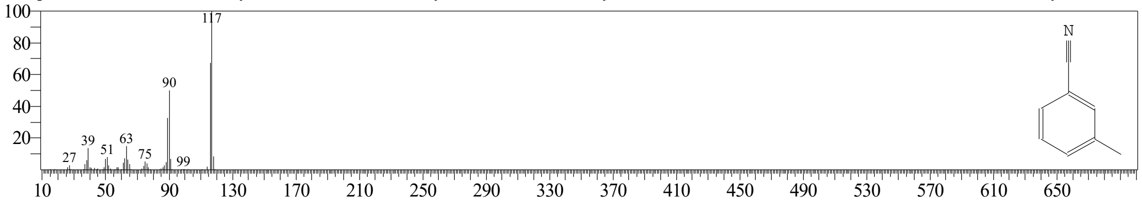

Hit#:5 Entry:4822 Library:NIST11.lib

SI:78 Formula:C8H7N CAS:529-19-1 MolWeight:117 RetIndex:1071

CompName:Benzonitrile, 2-methyl- $$ o-Tolunitrile $$ o-Cyanotoluene $$ o-Methylbenzonitrile $$ o-Toluonitrile $$ o-Tolylnitrile $$ 2-Cyanotoluene $$ 2-Me

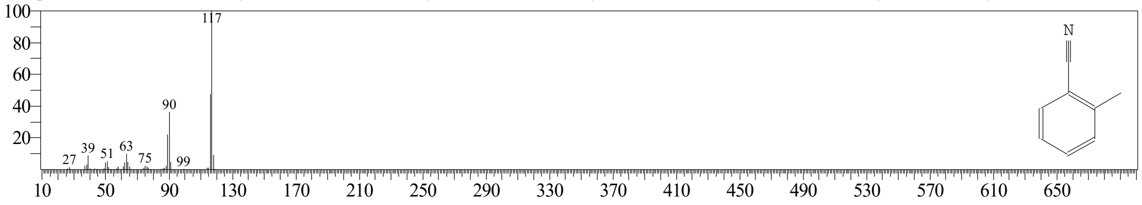

<< Target >>
Line#:2 R.Time:12.655(Scan#:1832) MassPeaks:374
RawMode:Averaged 12.650-12.660(1831-1833) BasePeak:100.05(9523)
BG Mode:Calc. from Peak Group 1 - Event 1 Scan

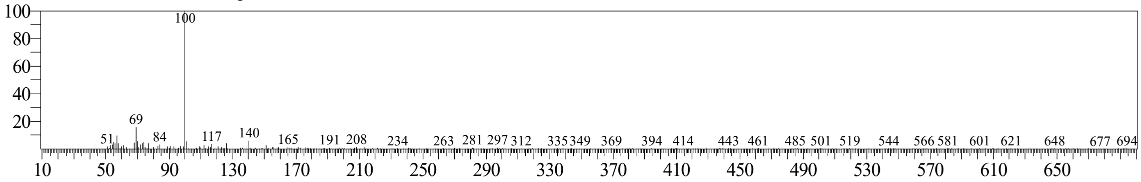

Hit#:1 Entry:142582 Library:NIST11.lib
SI:76 Formula:C13H17FN2O5S CAS:328010-30-6 MolWeight:332 RetIndex:2570
CompName:Morpholine, 4-[3-(4-fluoro-3-nitrophenylsulfonyl)propyl]- $$ 4-(3-[(4-Fluoro-3-nitrophenyl)sulfonyl]propyl)morpholine # $$

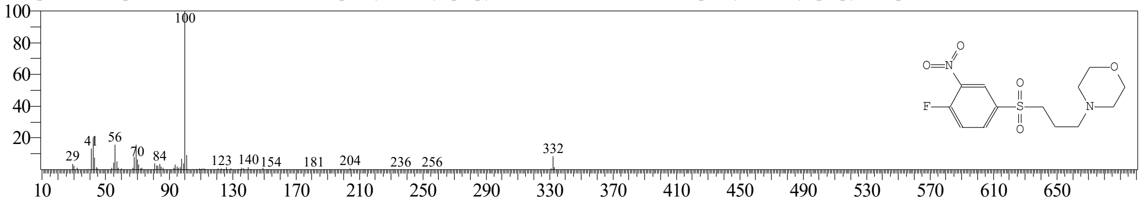

Hit#:2 Entry:1872 Library:NIST11.lib
SI:75 Formula:C3H8N4 CAS:0-00-0 MolWeight:100 RetIndex:1256
CompName:2-Hydrazino-2-imidazoline

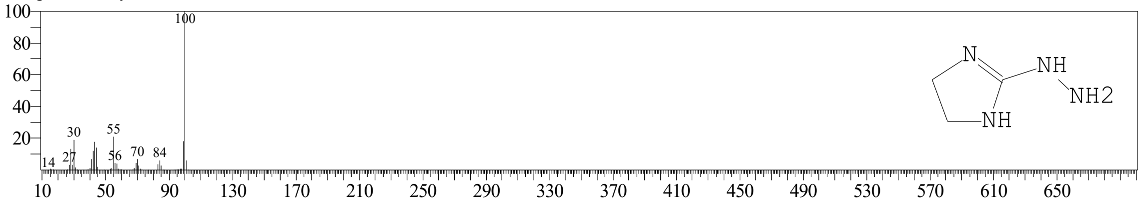

Hit#:3 Entry:7583 Library:NIST11.lib
SI:75 Formula:C6H16BNO CAS:149894-26-8 MolWeight:129 RetIndex:0
CompName:Ethylamine, 2-diethylboryloxy- $$ 2-Aminoethyl diethylborinate # $$

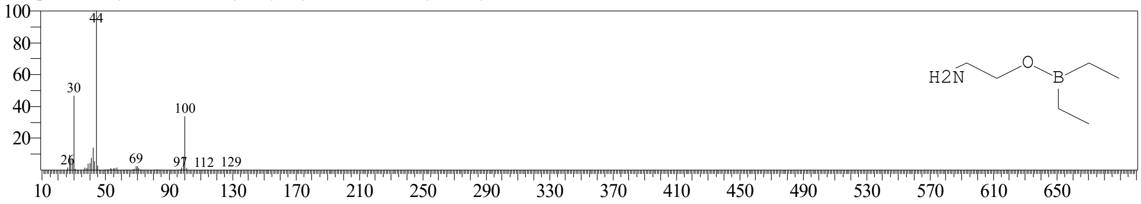

Hit#:4 Entry:82996 Library:NIST11.lib
SI:75 Formula:C10H17N5O3 CAS:0-00-0 MolWeight:255 RetIndex:2351
CompName:4-Amino-furazan-3-carboxylic acid (3-morpholin-4-yl-propyl)-amide $$ 4-Amino-N-[3-(4-morpholinyl)propyl]-1,2,5-oxadiazole-3-carboxamide # $

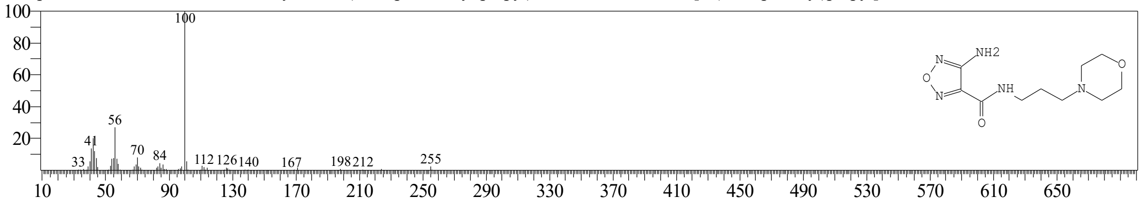

Hit#:5 Entry:64629 Library:NIST11.lib
SI:75 Formula:C10H18N2O4 CAS:342021-41-4 MolWeight:230 RetIndex:2075
CompName:N-[[2-Morpholino]ethyl]succinamic acid $$ 4-([2-(4-Morpholinyl)ethyl]amino)-4-oxobutanoic acid # $$

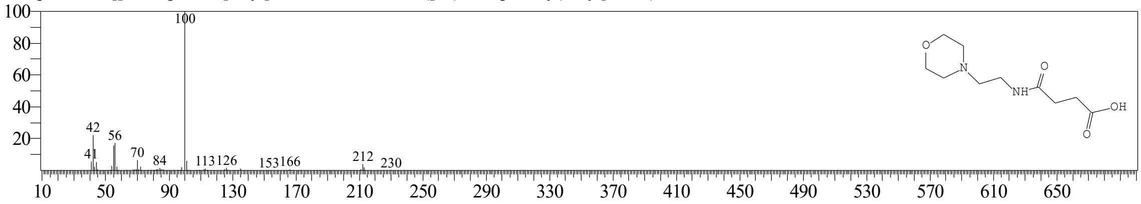

<< Target >>
Line#:3 R.Time:13.000(Scan#:1901) MassPeaks:316
RawMode:Averaged 12.995-13.005(1900-1902) BasePeak:60.05(3369)
BG Mode:Calc. from Peak Group 1 - Event 1 Scan

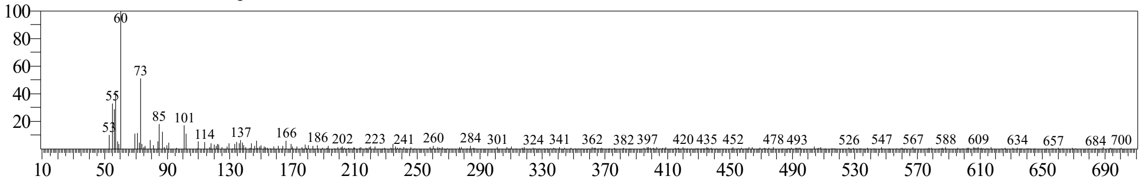

Hit#:1 Entry:12968 Library:NIST11.lib
SI:73 Formula:C8H16O2 CAS:124-07-2 MolWeight:144 RetIndex:1173
CompName:Octanoic acid $$ n-Caprylic acid $$ n-Octanoic acid $$ n-Octoic acid $$ n-Octylic acid $$ Neo-Fat 8 $$ Caprylic acid $$ Enantic acid $$ Octylic ac

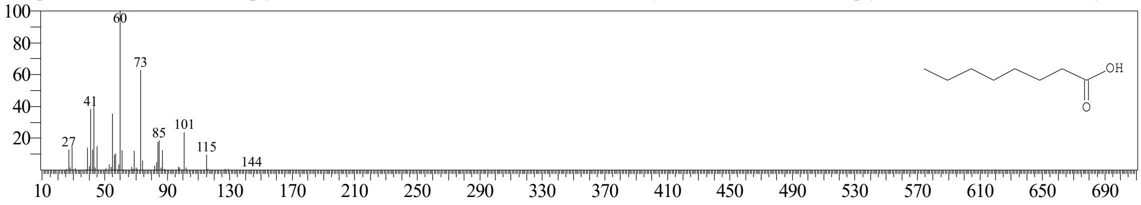

Hit#:2 Entry:30974 Library:NIST11.lib
SI:72 Formula:C6H12O6 CAS:2595-97-3 MolWeight:180 RetIndex:1698
CompName:D-Allose $$ .beta.-D-Allose $$ Hexose # $$

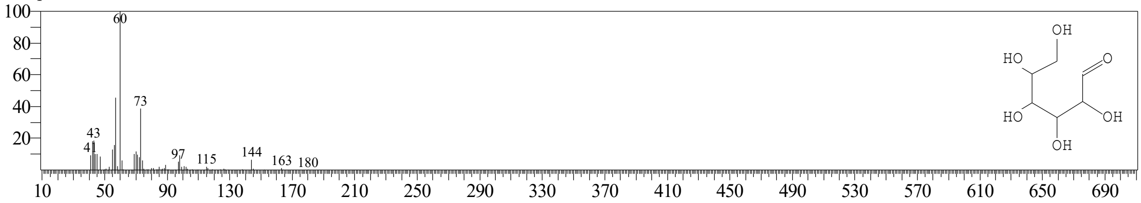

Hit#:3 Entry:20937 Library:NIST11.lib
SI:71 Formula:C6H10O5 CAS:0-00-0 MolWeight:162 RetIndex:1404
CompName:1,6-Anhydro-.beta.-d-talopyranose

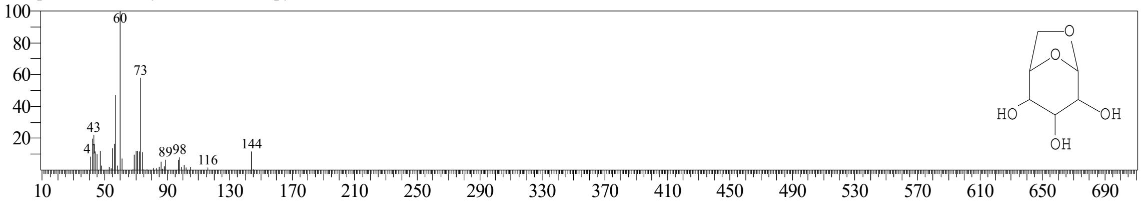

Hit#:4 Entry:212600 Library:NIST11.lib
SI:70 Formula:C38H60O18 CAS:77-05-4 MolWeight:804 RetIndex:6530
CompName:Stevioside $$ 1-O-(13-[(2-O-Hexopyranosylhexopyranosyl)oxy]-18-oxokaur-16-en-18-yl)hexopyranose # $$

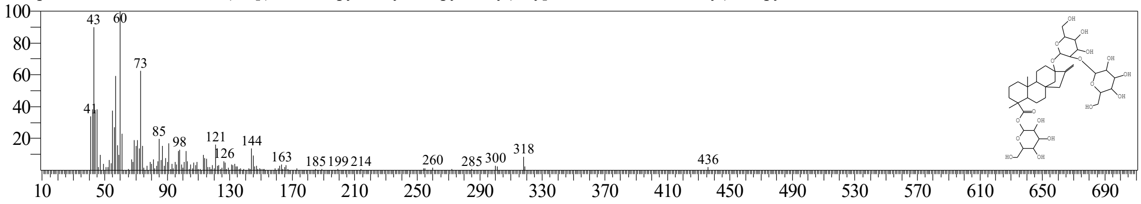

Hit#:5 Entry:27064 Library:NIST11.lib
SI:70 Formula:C10H20O2 CAS:334-48-5 MolWeight:172 RetIndex:1372
CompName:n-Decanoic acid $$ Decanoic acid $$ n-Capric acid $$ n-Decoic acid $$ n-Decylic acid $$ Capric acid $$ Caprinic acid $$ Caprynic acid $$ Decoic

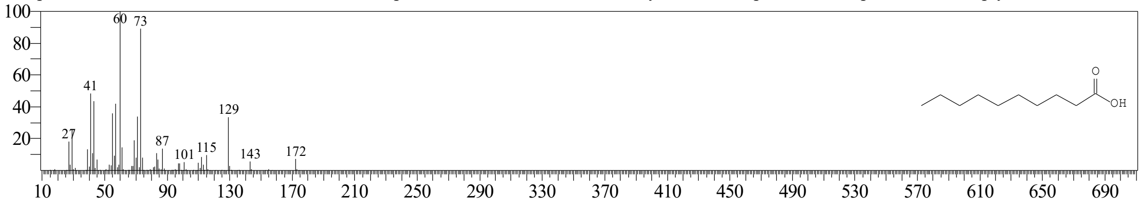

<< Target >>
Line#:4 R.Time:13.580(Scan#:2017) MassPeaks:366
RawMode:Averaged 13.575-13.585(2016-2018) BasePeak:100.05(1106)
BG Mode:Calc. from Peak Group 1 - Event 1 Scan

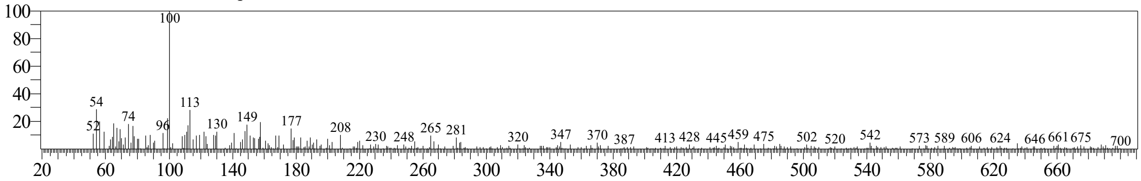

Hit#:1 Entry:44308 Library:NIST11.lib
SI:51 Formula:C9H15NO4 CAS:63535-65-9 MolWeight:201 RetIndex:1597
CompName:Acetate, (2-(3-hydroxy-3-methyl-2-oxotetrahydro-1H-1-pyrrolyl)ethyl] ester $$ 2-(3-Hydroxy-3-methyl-2-oxo-1-pyrrolidinyl)ethyl acetate # $$

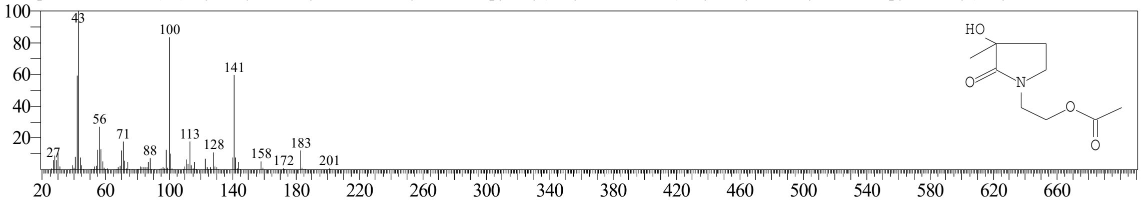

Hit#:2 Entry:7515 Library:NIST11.lib
SI:50 Formula:C5H8FN3 CAS:50581-18-5 MolWeight:129 RetIndex:1202
CompName:2-Fluorohistamine $$ 2-(2-Fluoro-1H-imidazol-5-yl)ethylamine # $$

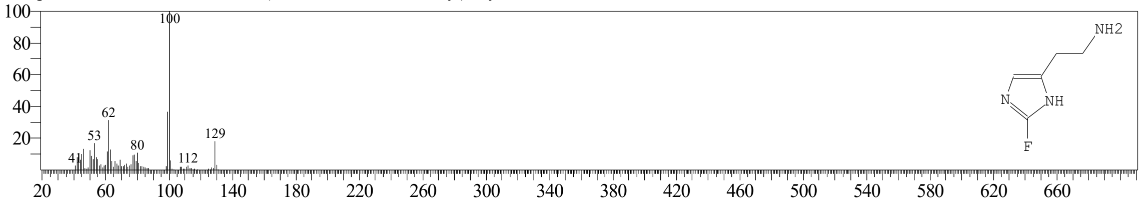

Hit#:3 Entry:27349 Library:NIST11.lib
SI:49 Formula:C6H8FN3O2 CAS:50444-78-5 MolWeight:173 RetIndex:1593
CompName:Histidine, 2-fluoro-, L- $$ 2FHis $$ 2-Fluorohistidine $$ L-2-Fluorohistidine $$ L-Histidine, 2-fluoro- $$ NSC 208713 $$

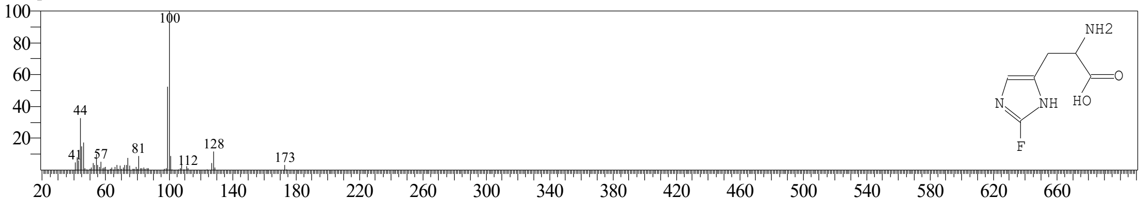

Hit#:4 Entry:116370 Library:NIST11.lib
SI:49 Formula:C13H18N2O4S CAS:0-00-0 MolWeight:298 RetIndex:2511
CompName:Thieno[3,4-b][1,4]dioxine-5-carboxylic acid, 2,3-dihydro-, (2-morpholin-4-ylethyl)amide

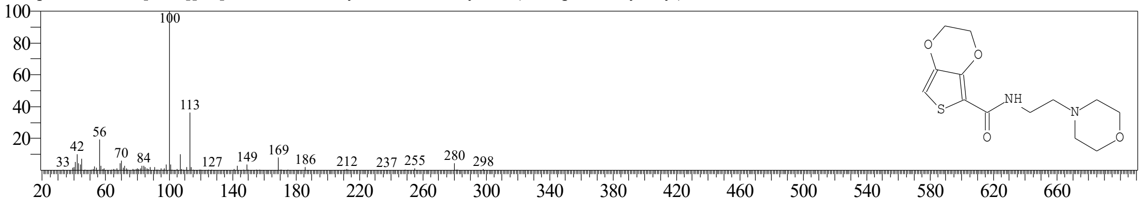

Hit#:5 Entry:51396 Library:NIST11.lib
SI:48 Formula:C11H21N3O CAS:0-00-0 MolWeight:211 RetIndex:1787
CompName:N-[6-[N-Aziridyl]-3-aza-3-hexenyl]morpholine $$ N-[(E)-3-(1-Aziridinyl)propylidene]-2-(4-morpholinyl)ethanamine # $$

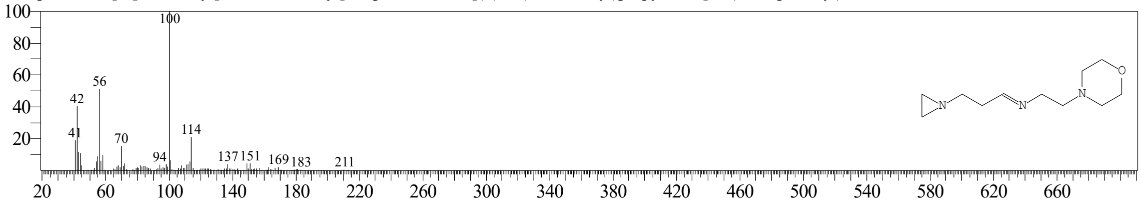

<< Target >>
Line#:5 R.Time:13.625(Scan#:2026) MassPeaks:345
RawMode:Averaged 13.620-13.630(2025-2027) BasePeak:100.05(10540)
BG Mode:Calc. from Peak Group 1 - Event 1 Scan

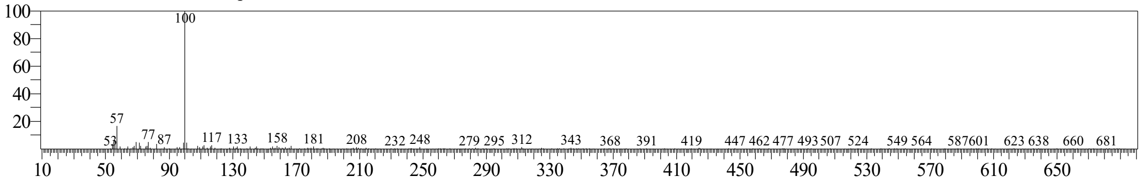

Hit#:1 Entry:1881 Library:NIST11.lib
SI:77 Formula:C4H8N2O CAS:1852-17-1 MolWeight:100 RetIndex:1028
CompName:N,N'-Trimethyleneurea $$ Tetrahydro-2-pyrimidone $$ 2(1H)-Pyrimidinone, tetrahydro- $$ Hexahydro-2(1H)-pyrimidinone $$ Hexahydropyrimidin-

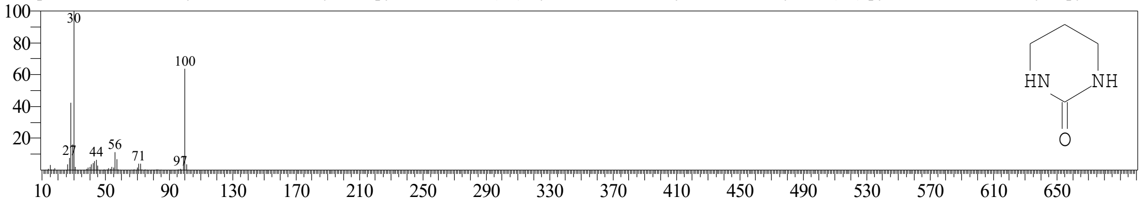

Hit#:2 Entry:18991 Library:NIST11.lib
SI:76 Formula:C10H23N CAS:2050-92-2 MolWeight:157 RetIndex:1214
CompName:1-Pentanamine, N-pentyl- $$ Dipentylamine $$ Di-n-amylamine $$ Di-n-pentylamine $$ Diamylamine $$ Pentylamine, pentyl- $$ UN 2841 $$ N,N-

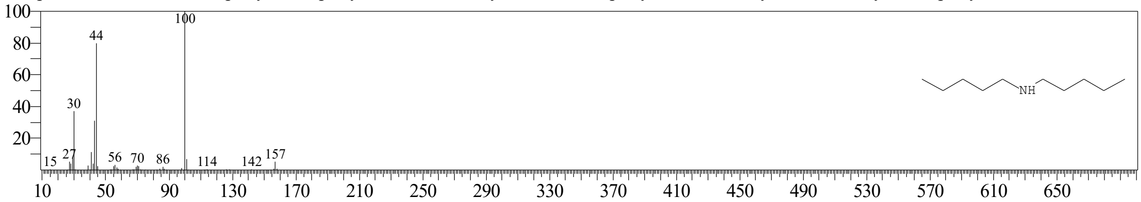

Hit#:3 Entry:7583 Library:NIST11.lib
SI:76 Formula:C6H16BNO CAS:149894-26-8 MolWeight:129 RetIndex:0
CompName:Ethylamine, 2-diethylboryloxy- $$ 2-Aminoethyl diethylborinate # $$

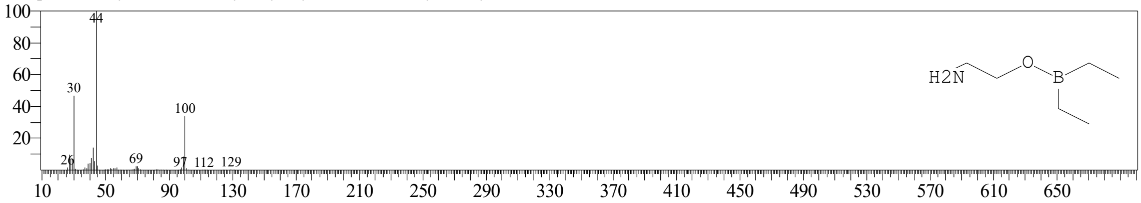

Hit#:4 Entry:148111 Library:NIST11.lib
SI:75 Formula:C22H45NO CAS:16528-77-1 MolWeight:339 RetIndex:2511
CompName:Morpholine, 4-octadecyl- $$ 4-Octadecylmorpholine # $$

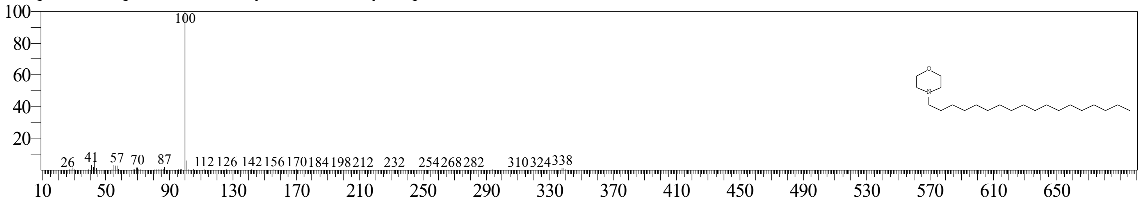

Hit#:5 Entry:26466 Library:NIST11.lib
SI:75 Formula:C11H25N CAS:33788-00-0 MolWeight:171 RetIndex:1294
CompName:6-Undecylamine $$ 6-Aminoundecane $$ 6-Undecanamine $$ 1-Pentylhexylamine $$

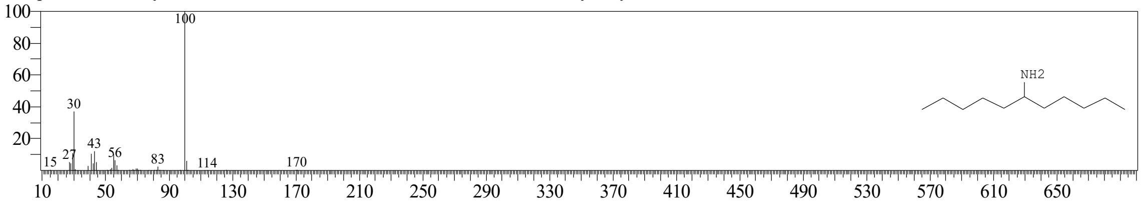

Qualitative Analysis Report

<< Target >>
Line#:6 R.Time:13.730(Scan#:2047) MassPeaks:321
RawMode:Averaged 13.725-13.735(2046-2048) BasePeak:100.05(788)
BG Mode:Calc. from Peak Group 1 - Event 1 Scan

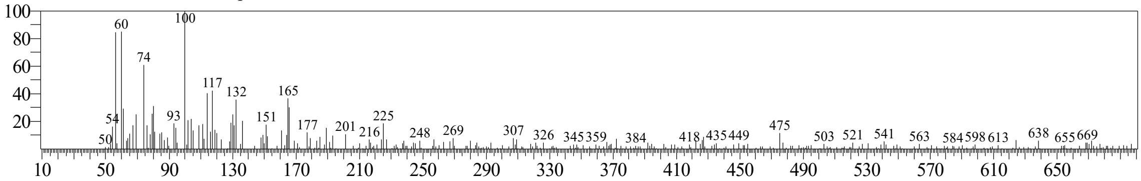

Hit#:1 Entry:61127 Library:NIST11.lib
SI:45 Formula:C10H11NO3S CAS:0-00-0 MolWeight:225 RetIndex:0
CompName:Thiazolo[3,2-a]pyridinium, 3-hydroxy-2-methyl-, acetate

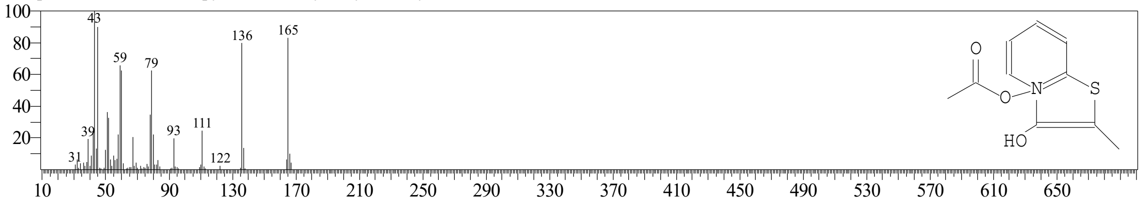

Hit#:2 Entry:8300 Library:NIST11.lib
SI:44 Formula:C4H8N2OS CAS:70629-19-5 MolWeight:132 RetIndex:1249
CompName:N-Nitroso-2-methylthiazolidine $$ Thiazolidine, 2-methyl-3-nitroso- $$ 2-Methyl-N-nitrosothiazolidine $$ 2-Methyl-3-nitroso-1,3-thiazolidine # $

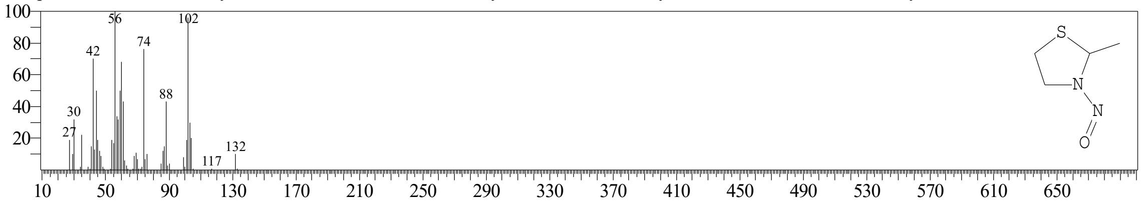

Hit#:3 Entry:29421 Library:NIST11.lib
SI:44 Formula:C7H15NO2S CAS:0-00-0 MolWeight:177 RetIndex:1314
CompName:2-Amino-4-methylthiobutyric acid, ethyl ester $$ Ethyl 2-amino-4-(methylsulfanyl)butanoate # $$

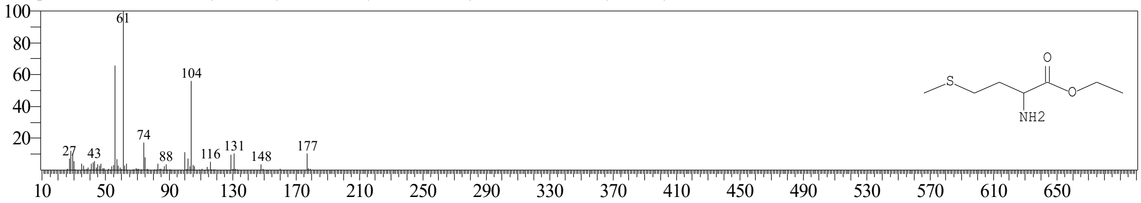

Hit#:4 Entry:22877 Library:NIST11.lib
SI:43 Formula:C10H15NO CAS:4500-12-3 MolWeight:165 RetIndex:1213
CompName:2-Adamantanone oxime $$ Tricyclo[3.3.1.1(3,7)-]decan-7-one, oxime $$ Adamantanone oxime $$ 2-Hydroxyiminoadamantane $$ Adamantanone-2-

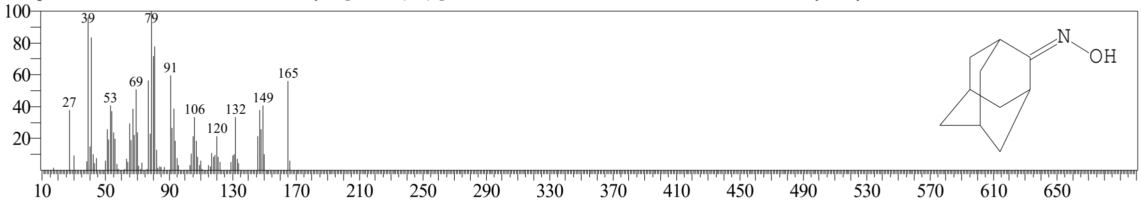

Hit#:5 Entry:52120 Library:NIST11.lib
SI:43 Formula:C12H20O3 CAS:135897-92-6 MolWeight:212 RetIndex:1594
CompName:8-Hydroxy-2,6-dimethylocta-2,6-dienoic acid, ethyl ester $$ Ethyl (2E,6Z)-8-hydroxy-2,6-dimethyl-2,6-octadienoate # $$

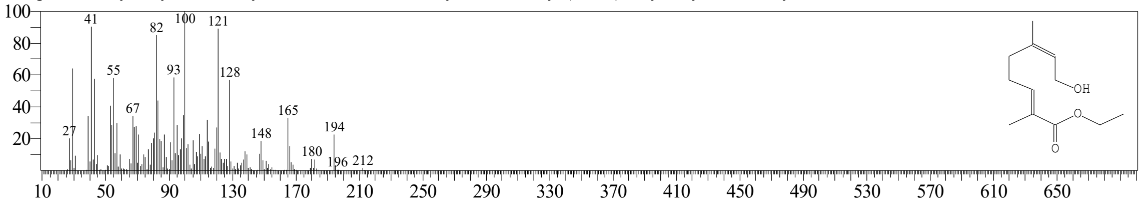

<< Target >>
Line#:7 R.Time:13.740(Scan#:2049) MassPeaks:352
RawMode:Averaged 13.735-13.745(2048-2050) BasePeak:83.05(975)
BG Mode:Calc. from Peak Group 1 - Event 1 Scan

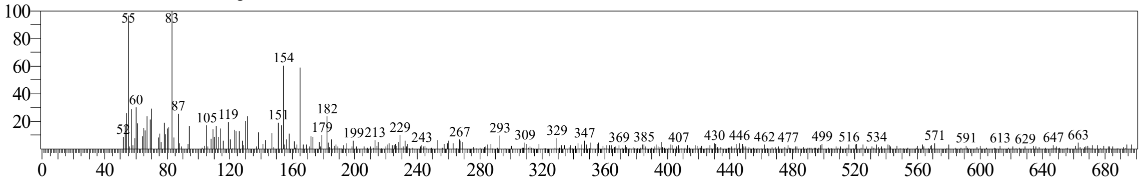

Hit#:1 Entry:49649 Library:NIST11.lib
SI:57 Formula:C15H28 CAS:50991-15-6 MolWeight:208 RetIndex:1537
CompName:1,1'-Bicyclohexyl, 2-(1-methylethyl)-, cis- $$ Bicyclohexyl, 2-isopropyl-, cis- $$

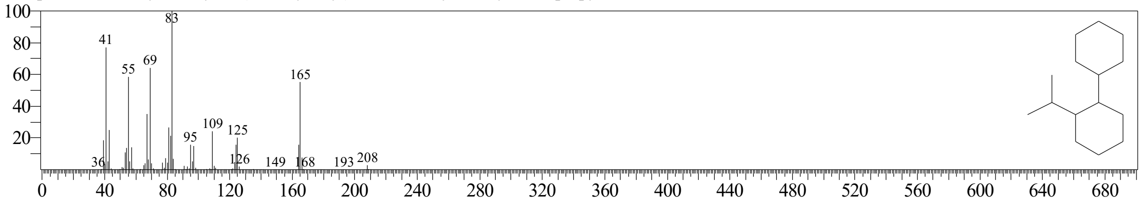

Hit#:2 Entry:49650 Library:NIST11.lib
SI:57 Formula:C15H28 CAS:50991-16-7 MolWeight:208 RetIndex:1537
CompName:1,1'-Bicyclohexyl, 2-(1-methylethyl)-, trans- $$ Bicyclohexyl, 2-isopropyl-, trans- $$

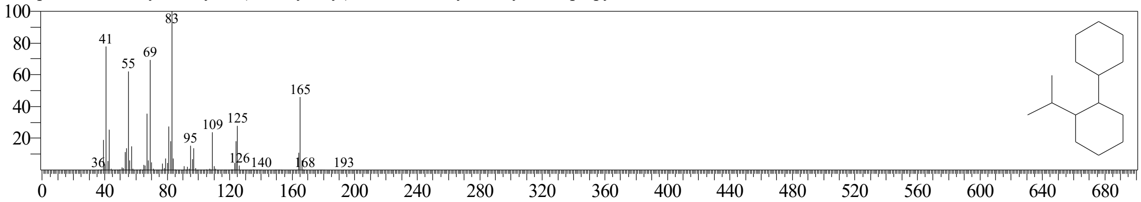

Hit#:3 Entry:79807 Library:NIST11.lib
SI:56 Formula:C16H31BO CAS:61142-73-2 MolWeight:250 RetIndex:0
CompName:Borinic acid, diethyl-, 1-cyclododecen-1-yl ester $$ 1-Cyclododecen-1-yl diethylborinate # $$

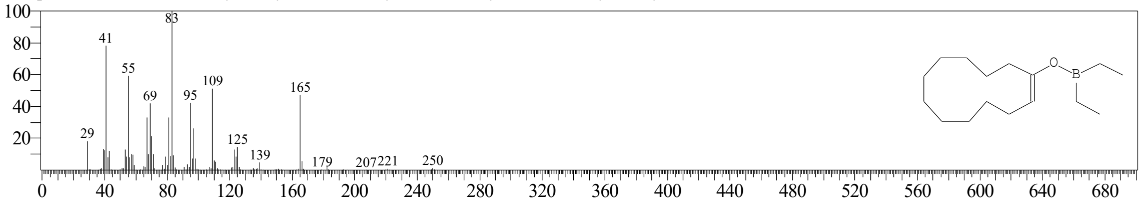

Hit#:4 Entry:17433 Library:NIST11.lib
SI:56 Formula:C10H18O CAS:14507-02-9 MolWeight:154 RetIndex:1274
CompName:2,4-Decadien-1-ol $$ 2,4-Decadienol $$

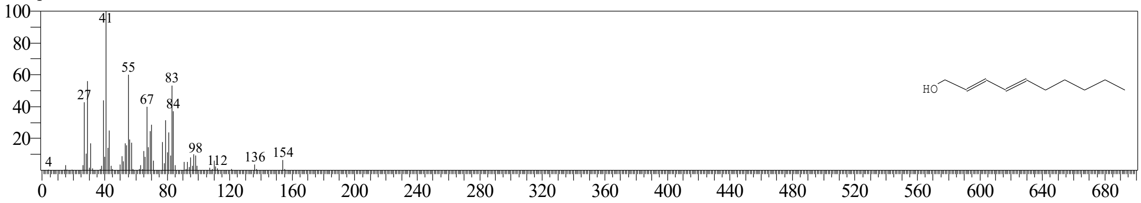

Hit#:5 Entry:17582 Library:NIST11.lib
SI:56 Formula:C10H18O CAS:0-00-0 MolWeight:154 RetIndex:1233
CompName:2,4-Pentadien-1-ol, 3-pentyl-, (2Z)- $$ (2E)-3-Pentyl-2,4-pentadien-1-ol # $$

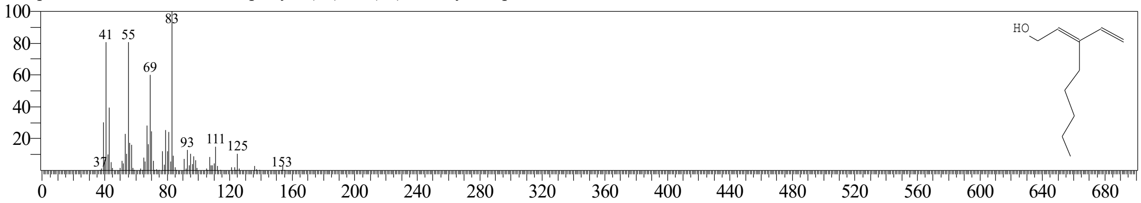

<< Target >>
Line#:8 R.Time:14.435(Scan#:2188) MassPeaks:366
RawMode:Averaged 14.430-14.440(2187-2189) BasePeak:81.05(4723)
BG Mode:Calc. from Peak Group 1 - Event 1 Scan

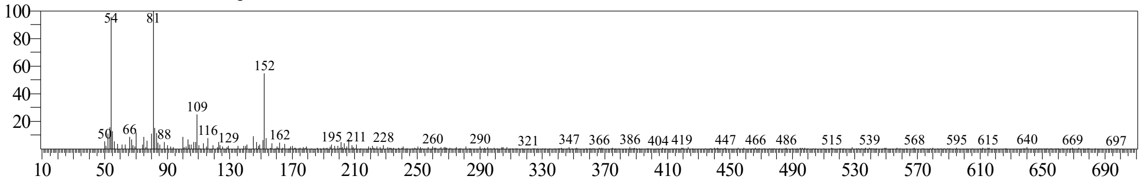

Hit#:1 Entry:15900 Library:NIST11.lib
SI:67 Formula:C5H4N4O2 CAS:69-89-6 MolWeight:152 RetIndex:1641
CompName:Xanthine $$ 1H-Purine-2,6-dione, 3,7-dihydro- $$ Isoxanthine $$ Pseudoxanthine $$ Purine-2,6(1H,3H)-dione $$ Xan $$ Xanthic oxide $$ Xanthi

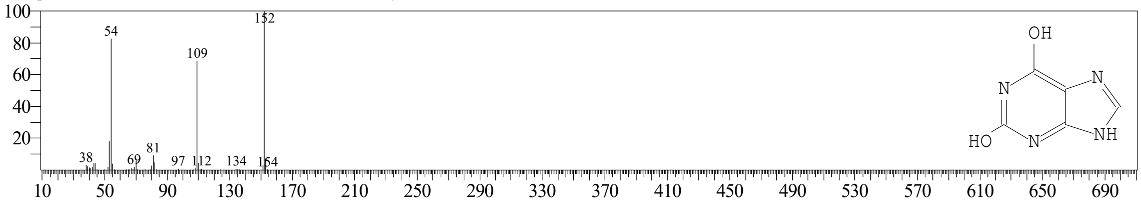

Hit#:2 Entry:2937 Library:NIST11.lib
SI:66 Formula:C5H7N3 CAS:0-00-0 MolWeight:109 RetIndex:1159
CompName:(2H)Pyrrole-2-carbonitrile, 5-amino-3,4-dihydro- $$ 5-Amino-3,4-dihydro-2H-pyrrole-2-carbonitrile # $$

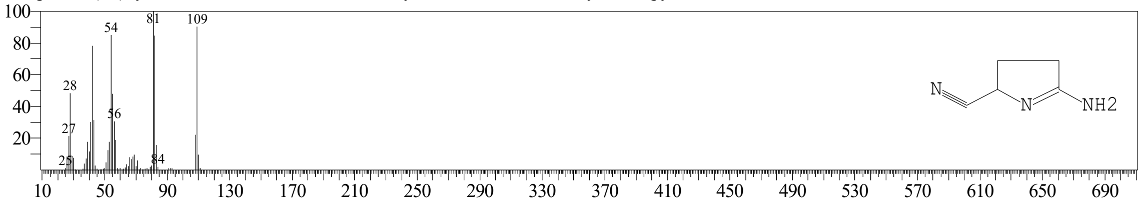

Hit#:3 Entry:16014 Library:NIST11.lib
SI:66 Formula:C7H8N2O2 CAS:17384-56-4 MolWeight:152 RetIndex:1577
CompName:1H-Pyrrolo[2,3-b]pyridine-2,6-dione, 3,3a,4,5-tetrahydro- $$ 3,3a,4,5-Tetrahydro-1H-pyrrolo[2,3-b]pyridine-2,6-dione # $$

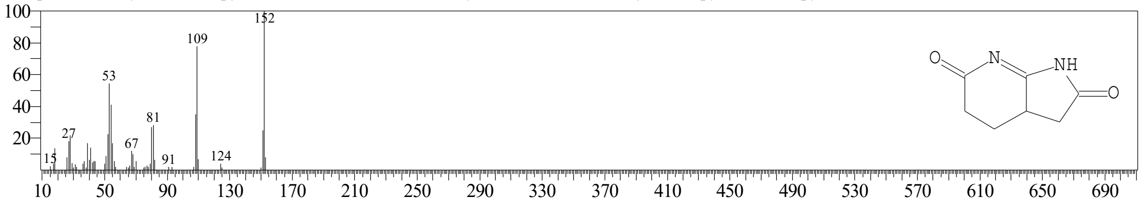

Hit#:4 Entry:16393 Library:NIST11.lib
SI:65 Formula:C10H16O CAS:15932-80-6 MolWeight:152 RetIndex:1212
CompName:Cyclohexanone, 5-methyl-2-(1-methylethylidene)- $$ p-Menth-4(8)-en-3-one $$ (.+/-.)-Pulegone $$ 2-Isopropylidene-5-methylcyclohexanone $$ 4(

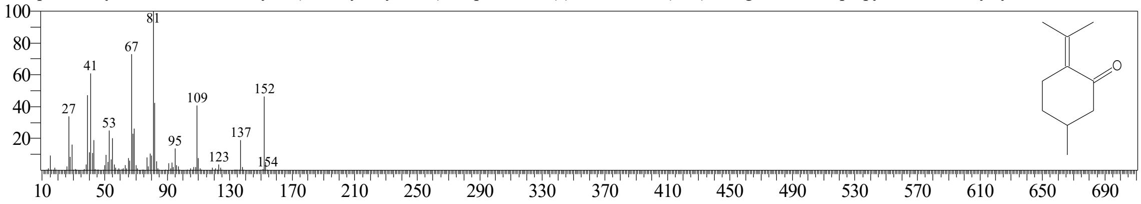

Hit#:5 Entry:16190 Library:NIST11.lib
SI:65 Formula:C9H12O2 CAS:58512-56-4 MolWeight:152 RetIndex:1041
CompName:2-Oxaadamantan-6-one

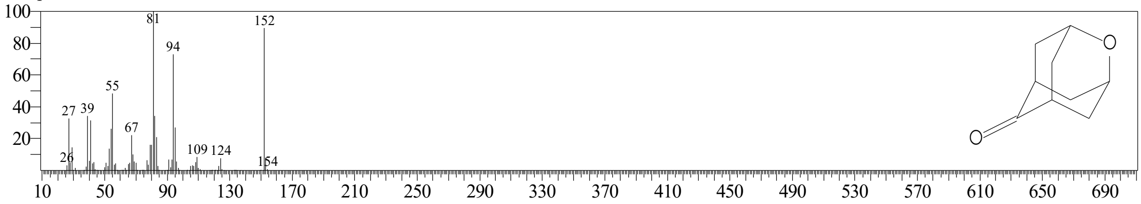

<< Target >>
Line#:9 R.Time:14.485(Scan#:2198) MassPeaks:373
RawMode:Averaged 14.480-14.490(2197-2199) BasePeak:81.10(1259)
BG Mode:Calc. from Peak Group 1 - Event 1 Scan

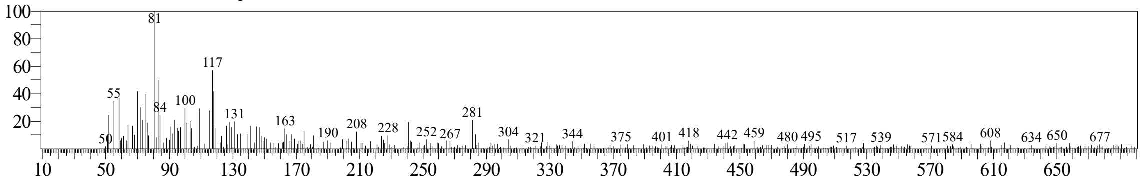

Hit#:1 Entry:169344 Library:NIST11.lib
SI:54 Formula:C25H40O2 CAS:0-00-0 MolWeight:372 RetIndex:2670
CompName:4-Hexyl-1-(7-methoxycarbonylheptyl)bicyclo[4.4.0]deca-2,5,7-triene $$ Methyl 8-(7-hexyl-3,7-dihydro-4a(4H)-naphthalenyl)octanoate # $$

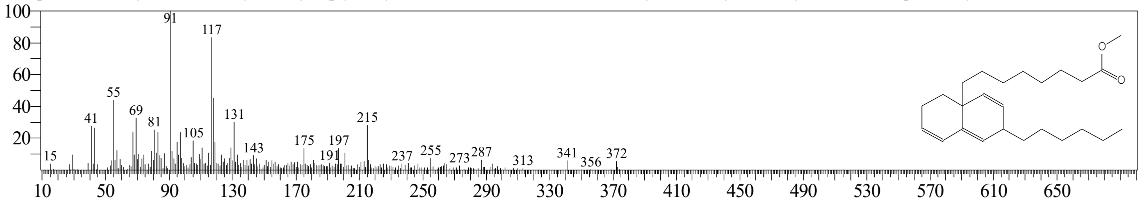

Hit#:2 Entry:149864 Library:NIST11.lib
SI:52 Formula:C15H29F3O3Si CAS:0-00-0 MolWeight:342 RetIndex:1436
CompName:1-Decanol, 9-[(trimethylsilyl)oxy]-, trifluoroacetate $$ 9-[(Trimethylsilyl)oxy]decyl trifluoroacetate # $$

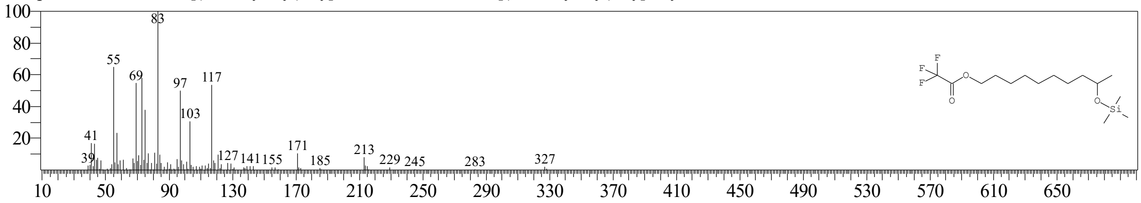

Hit#:3 Entry:30269 Library:NIST11.lib
SI:50 Formula:C11H14O2 CAS:70220-91-6 MolWeight:178 RetIndex:1170
CompName:10-Methoxytricyclo[4.2.1.1(2,5)]deca-3,7-dien-9-ol

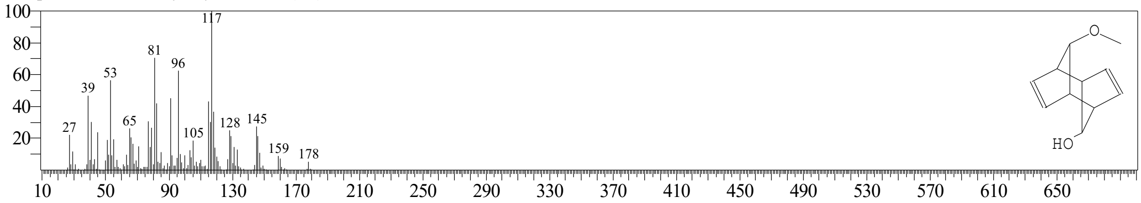

Hit#:4 Entry:99576 Library:NIST11.lib
SI:50 Formula:C14H29FO2Si CAS:26305-97-5 MolWeight:276 RetIndex:1463
CompName:Undecanoic acid, 11-fluoro-, trimethylsilyl ester $$ Trimethylsilyl 11-fluoroundecanoate # $$

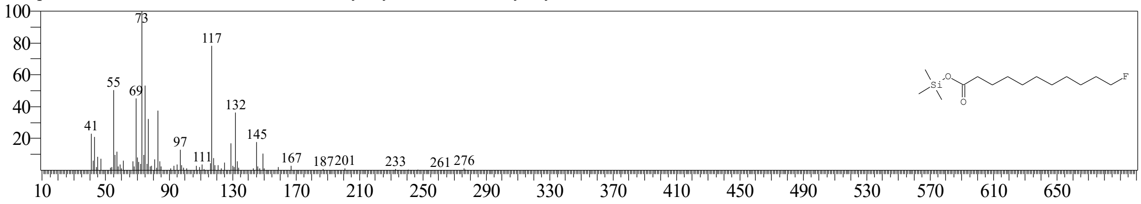

Hit#:5 Entry:166663 Library:NIST11.lib
SI:50 Formula:C22H44O2Si CAS:0-00-0 MolWeight:368 RetIndex:2270
CompName:12-Methyloctadec-11-enoic acid trimethylsilyl ester

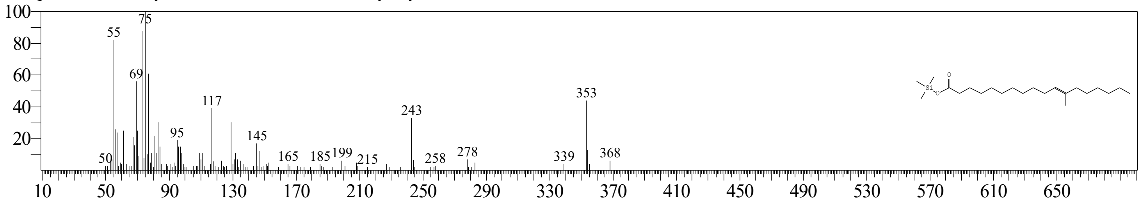

Qualitative Analysis Report

<< Target >>
Line#:10 R.Time:14.520(Scan#:2205) MassPeaks:352
RawMode:Averaged 14.515-14.525(2204-2206) BasePeak:54.10(1077)
BG Mode:Calc. from Peak Group 1 - Event 1 Scan

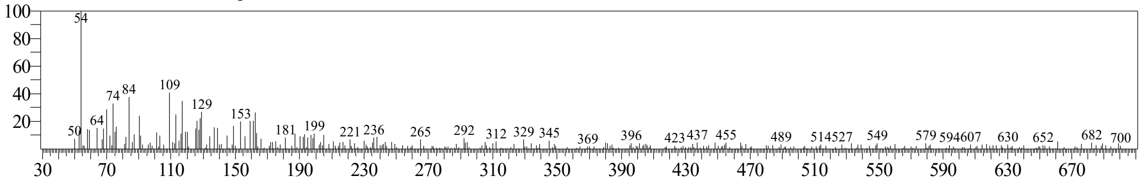

Hit#:1 Entry:120564 Library:NIST11.lib
SI:47 Formula:C13H21NO7 CAS:35439-79-3 MolWeight:303 RetIndex:1865
CompName:2,5-Di-O-acetyl-3,4,6-tri-O-methyl-D-gluconitrile

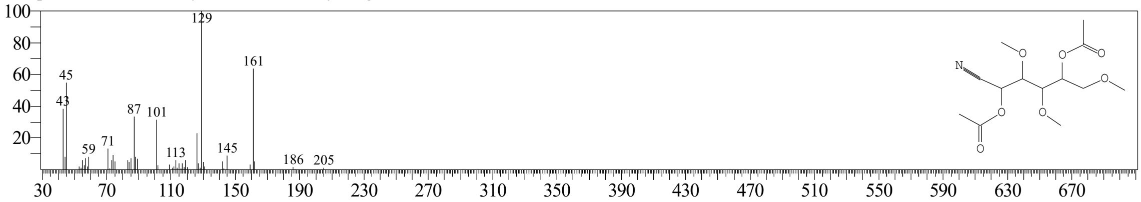

Hit#:2 Entry:100735 Library:NIST11.lib
SI:46 Formula:C12H22O7 CAS:84925-40-6 MolWeight:278 RetIndex:1584
CompName:1,4-Di-O-acetyl-2,3,5-tri-O-methylribitol $$ 1,4-Di-O-acetyl-2,3,5-tri-O-methylpentitol # $$

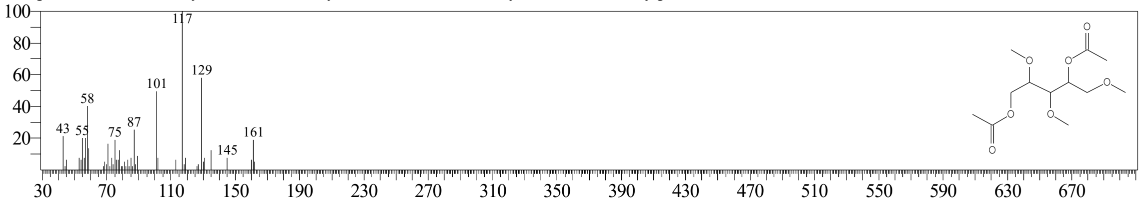

Hit#:3 Entry:20883 Library:NIST11.lib
SI:44 Formula:C5H4F2N2O2 CAS:0-00-0 MolWeight:162 RetIndex:1045
CompName:5H-Pyrimidine-2,4-dione, 5,6-difluoro-5-methyl-

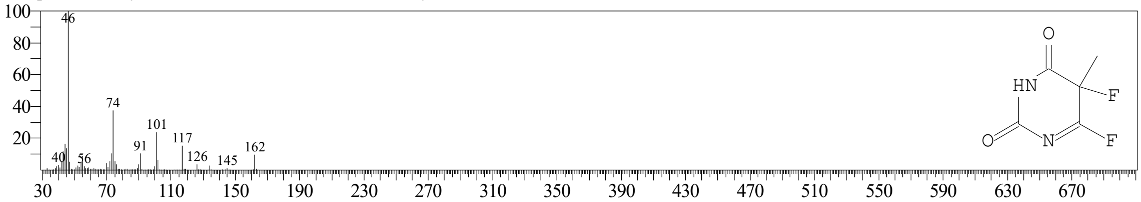

Hit#:4 Entry:172058 Library:NIST11.lib
SI:44 Formula:C16H26O10 CAS:84582-64-9 MolWeight:378 RetIndex:2276
CompName:Methyl 3,4,7-tri-O-acetyl-2,6-di-O-methyl-.beta.-glycero-d-glucoheptopyranoside $$ Methyl 3,4,7-tri-O-acetyl-2,6-di-O-methylheptopyranoside # $

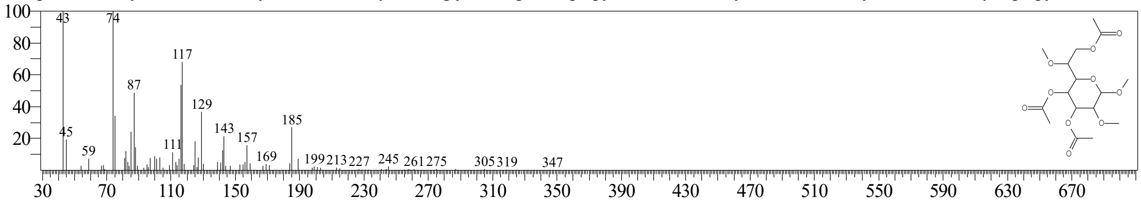

Hit#:5 Entry:12749 Library:NIST11.lib
SI:43 Formula:C6H12N2O2 CAS:55401-87-1 MolWeight:144 RetIndex:1199
CompName:Hydrazinecarboxylic acid, butylidene-, methyl ester $$ Methyl (2E)-2-butylidenehydrazinecarboxylate # $$

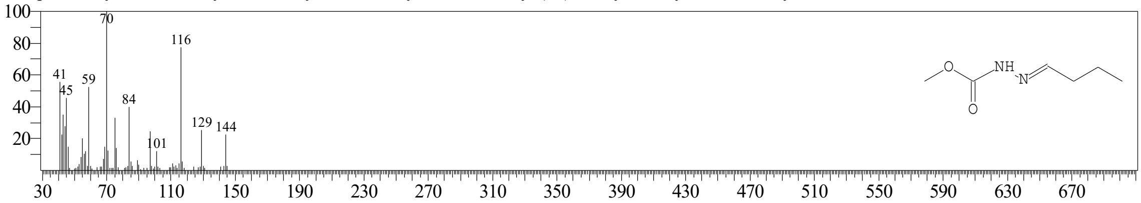

<< Target >>
Line#:11 R.Time:14.560(Scan#:2213) MassPeaks:346
RawMode:Averaged 14.555-14.565(2212-2214) BasePeak:59.05(499)
BG Mode:Calc. from Peak Group 1 - Event 1 Scan

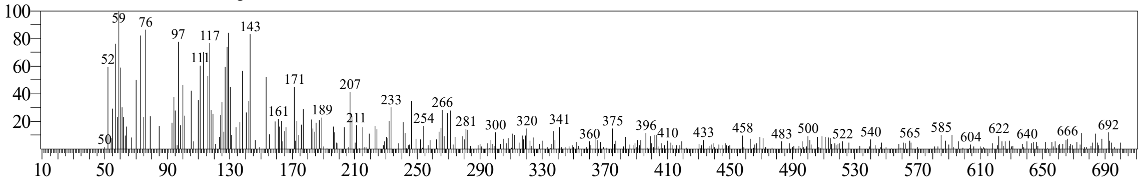

Hit#:1 Entry:190849 Library:NIST11.lib
SI:44 Formula:C24H54Si3 CAS:89463-49-0 MolWeight:426 RetIndex:1576
CompName:Hexa-t-butylcyclotrisilane $$ 1,1,2,2,3,3-Hexatert-butyltrisilirane # $$

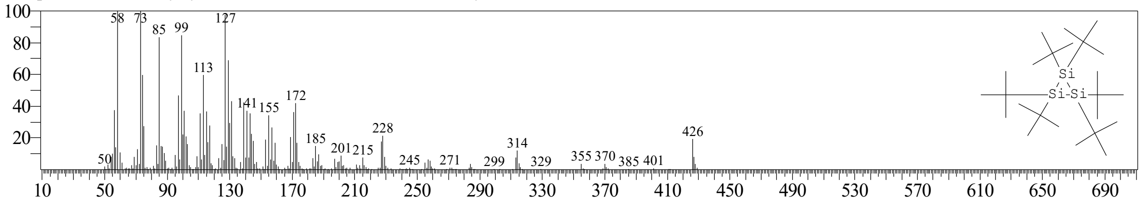

Hit#:2 Entry:121268 Library:NIST11.lib
SI:44 Formula:C14H28O5Si CAS:85951-12-8 MolWeight:304 RetIndex:1710
CompName:.alpha.-D-Xylofuranose, 1,2-O-isopropylidene-5-(t-butyldimethylsilyl)- $$ 5-O-[tert-Butyl(dimethyl)silyl]-1,2-O-(1-methylethylidene)pentofuranos

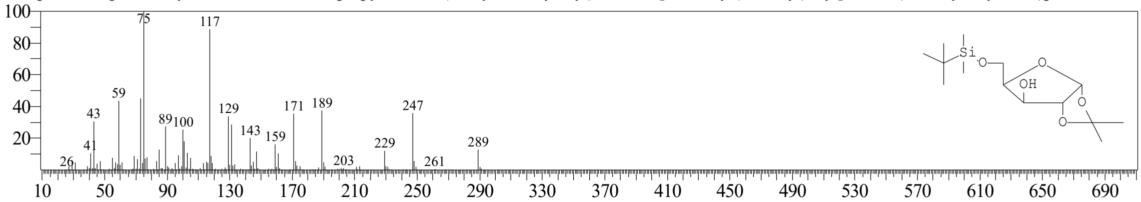

Hit#:3 Entry:127925 Library:NIST11.lib
SI:43 Formula:C18H36O2Si CAS:0-00-0 MolWeight:312 RetIndex:1938
CompName:n-Tridecanoic acid,methyl(tetramethylene)silyl ester $$ 1-Methyl-1-silolanyl tridecanoate # $$

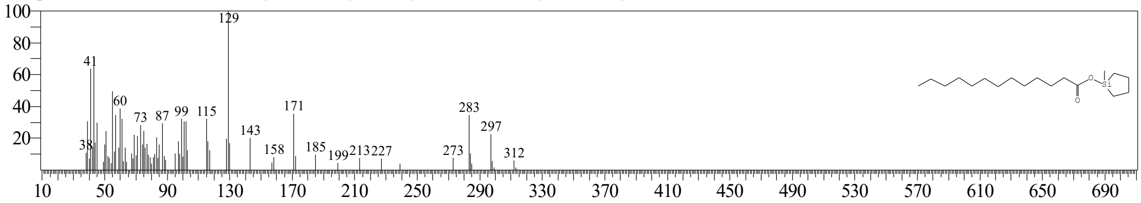

Hit#:4 Entry:95361 Library:NIST11.lib
SI:43 Formula:C7H9N7OS2 CAS:0-00-0 MolWeight:271 RetIndex:2637
CompName:2-(4-Amino-5-thioxo-4,5-dihydro-[1,2,4]triazol-1-yl)-N-(5-methyl-[1,3,4]thiadiazol-2-yl)-acetamide $$ 2-(4-Amino-5-thioxo-4,5-dihydro-1H-1,2,4-

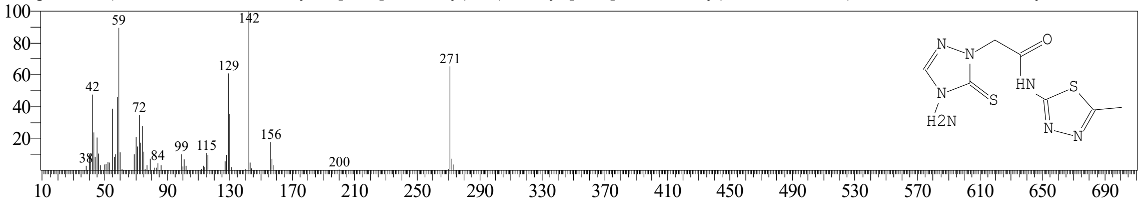

Hit#:5 Entry:85333 Library:NIST11.lib
SI:43 Formula:C12H18O6 CAS:55030-37-0 MolWeight:258 RetIndex:1821
CompName:Decanedioic acid, 3,8-dioxo-, dimethyl ester $$ Dimethyl 3,8-dioxodecanedioate # $$

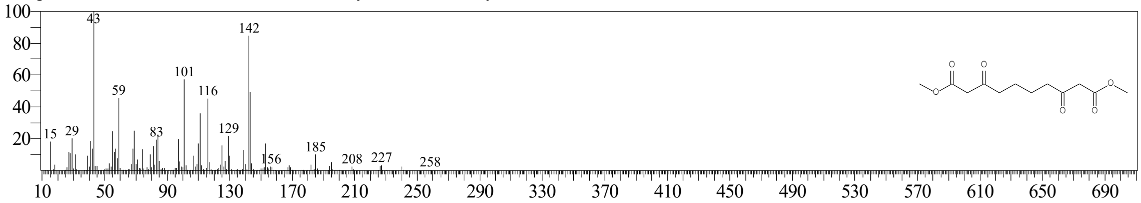

Qualitative Analysis Report

<< Target >>
Line#:12 R.Time:14.605(Scan#:2222) MassPeaks:357
RawMode:Averaged 14.600-14.610(2221-2223) BasePeak:60.10(846)
BG Mode:Calc. from Peak Group 1 - Event 1 Scan

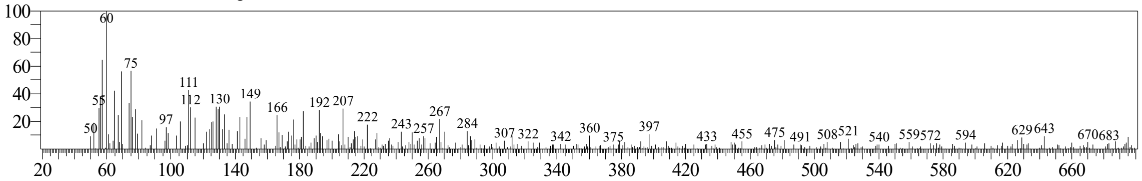

Hit#:1 Entry:115418 Library:NIST11.lib
SI:47 Formula:C19H36O2 CAS:108708-61-8 MolWeight:296 RetIndex:2082
CompName:Methyl trans-9-(2-butylcyclopentyl)nonanoate $$ Methyl 9-(2-butylcyclopentyl)nonanoate # $$

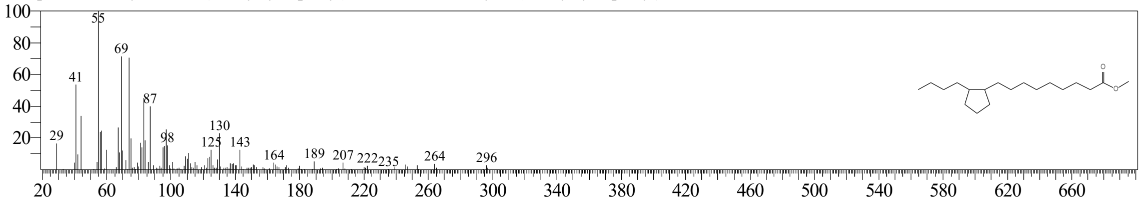

Hit#:2 Entry:73841 Library:NIST11.lib
SI:46 Formula:C15H30O2 CAS:10580-24-2 MolWeight:242 RetIndex:1680
CompName:n-Butyl n-undecanoate $$ Butyl undecanoate # $$

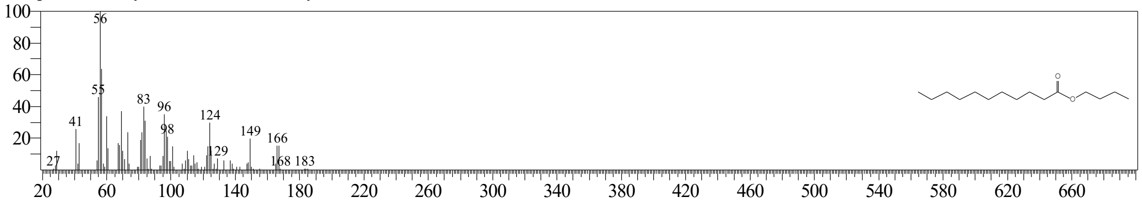

Hit#:3 Entry:150695 Library:NIST11.lib
SI:45 Formula:C14H17NO9 CAS:0-00-0 MolWeight:343 RetIndex:2271
CompName:Tetraacetyl-d-xylonic nitrile

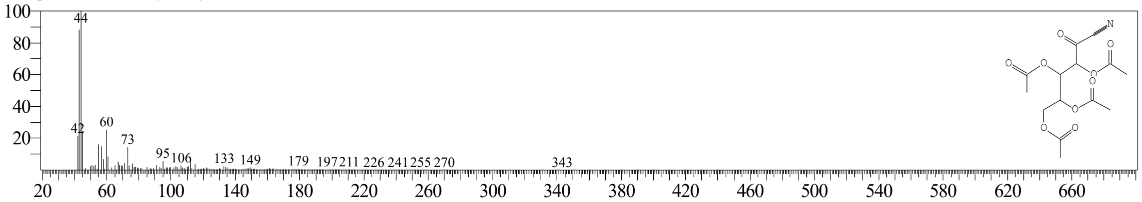

Hit#:4 Entry:28374 Library:NIST11.lib
SI:45 Formula:C7H13NO4 CAS:627-76-9 MolWeight:175 RetIndex:1609
CompName:dl-2-Aminopimelic acid $$ dl-.alpha.-Aminopimelic acid $$ Heptanedioic acid, 2-amino-, (.+/-.)- $$ 2-Aminoheptanedioic acid # $$ DL-2-Aminopi

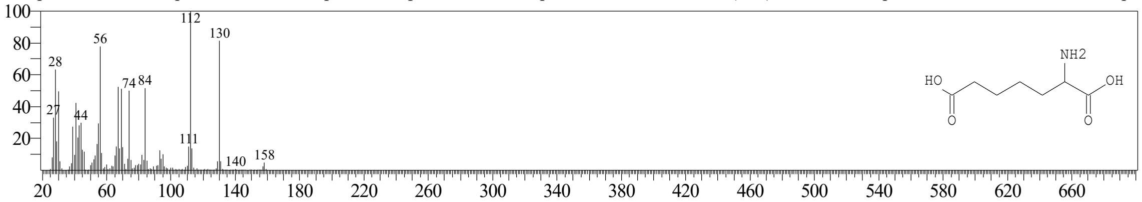

Hit#:5 Entry:160780 Library:NIST11.lib
SI:45 Formula:C21H42O4 CAS:621-61-4 MolWeight:358 RetIndex:2697
CompName:Octadecanoic acid, 2-hydroxy-1-(hydroxymethyl)ethyl ester $$ Stearin, 2-mono- $$ .beta.-Glyceryl monostearate $$ .beta.-Monostearin $$ Glycerol-

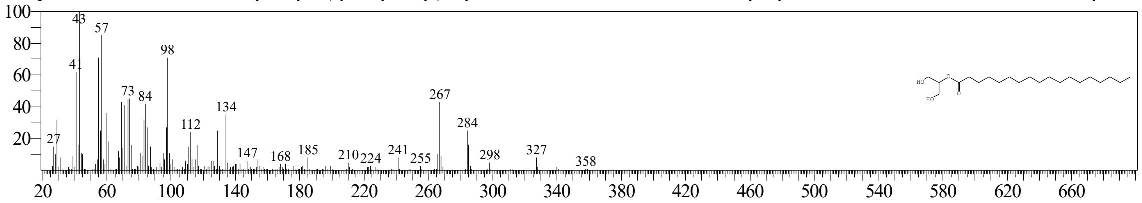

<< Target >>
Line#:13 R.Time:14.690(Scan#:2239) MassPeaks:340
RawMode:Averaged 14.685-14.695(2238-2240) BasePeak:129.10(520)
BG Mode:Calc. from Peak Group 1 - Event 1 Scan

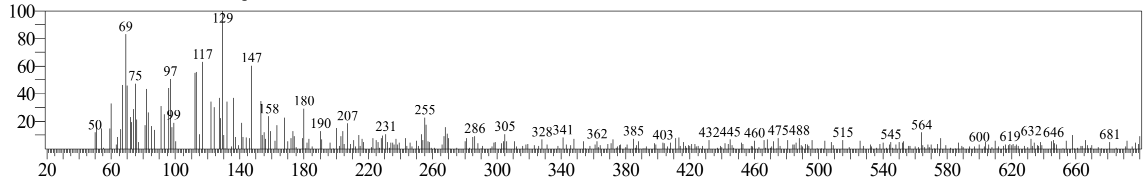

Hit#:1 Entry:35978 Library:NIST11.lib
SI:49 Formula:C9H16O4 CAS:0-00-0 MolWeight:188 RetIndex:1375
CompName:3-Propylglutaric acid, monomethyl ester $$ 3-(2-Methoxy-2-oxoethyl)hexanoic acid # $$

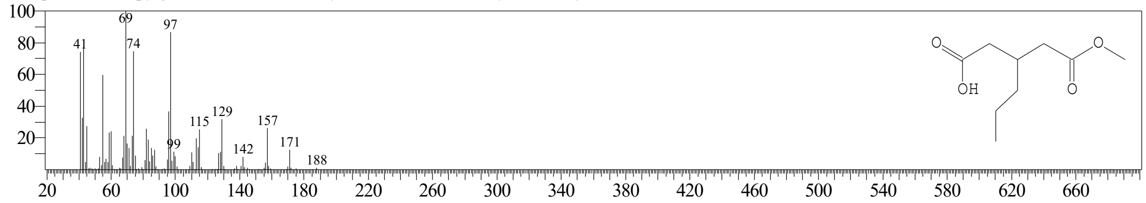

Hit#:2 Entry:18391 Library:NIST11.lib
SI:49 Formula:C9H16O2 CAS:91531-44-1 MolWeight:156 RetIndex:1115
CompName:1-(1-Methoxycyclopropyl)-3-methylbut-2-en-1-ol $$ 1-(1-Methoxycyclopropyl)-3-methyl-2-buten-1-ol # $$

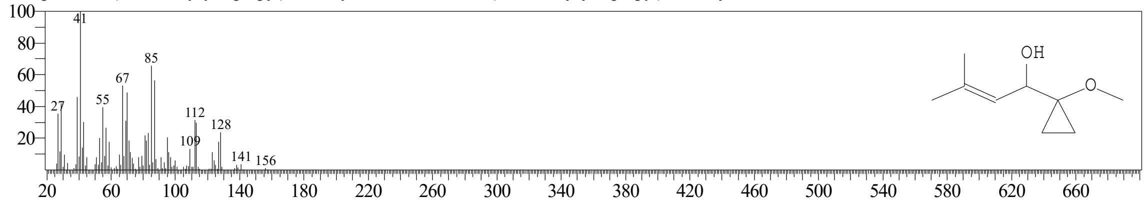

Hit#:3 Entry:137885 Library:NIST11.lib
SI:47 Formula:C13H14N2O6S CAS:39687-12-2 MolWeight:326 RetIndex:2367
CompName:6H-Furo[2',3':4,5]oxazolo[3,2-a]pyrimidine-6-thione, 3-(acetyloxy)-2-[(acetyloxy)methyl]-2,3,3a,9a-tetrahydro-, [2R-(2.alpha.,3.beta.,3a.beta.,9a.be

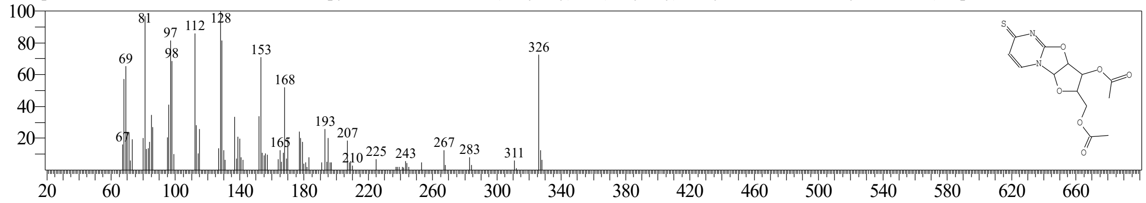

Hit#:4 Entry:44867 Library:NIST11.lib
SI:47 Formula:C10H18O4 CAS:14226-72-3 MolWeight:202 RetIndex:1286
CompName:Heptanedioic acid, 3-methyl-, dimethyl ester, (+)- $$ Dimethyl 3-methylheptanedioate # $$

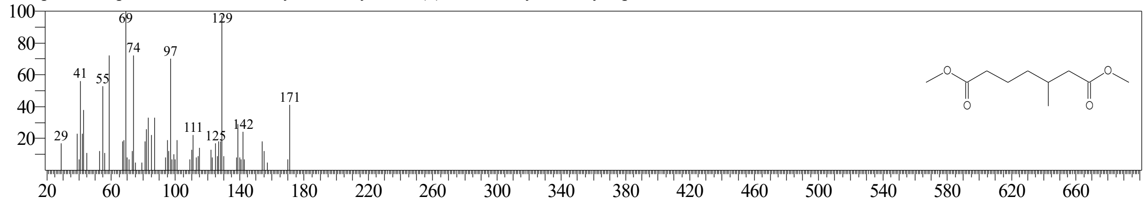

Hit#:5 Entry:7113 Library:NIST11.lib
SI:46 Formula:C7H12O2 CAS:50652-78-3 MolWeight:128 RetIndex:828
CompName:2-Pentenoic acid, 4-methyl-, methyl ester $$ Methyl 4-methyl-2-pentenoate $$ Methyl (2E)-4-methyl-2-pentenoate # $$

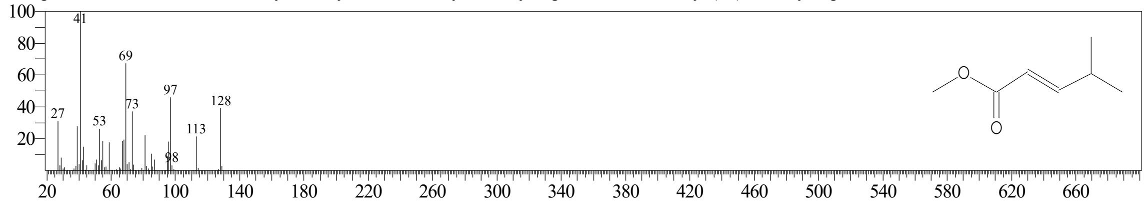

<< Target >>
Line#:14 R.Time:14.795(Scan#:2260) MassPeaks:352
RawMode:Averaged 14.790-14.800(2259-2261) BasePeak:71.15(664)
BG Mode:Calc. from Peak Group 1 - Event 1 Scan

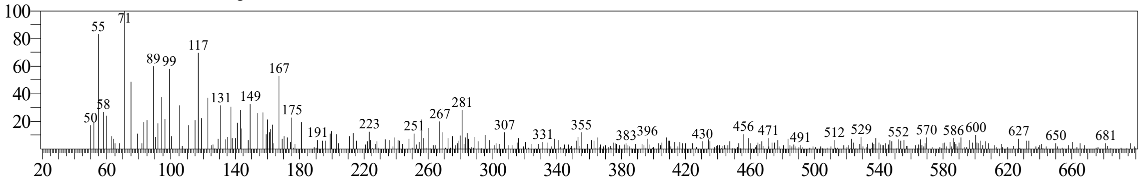

Hit#:1 Entry:57154 Library:NIST11.lib
SI:46 Formula:C10H20O5 CAS:55887-72-4 MolWeight:220 RetIndex:1310
CompName:D-Galactitol, 3,6-anhydro-1,2,4,5-tetra-O-methyl- $$ 3,6-Anhydro-1,2,4,5-tetra-O-methylhexitol # $$

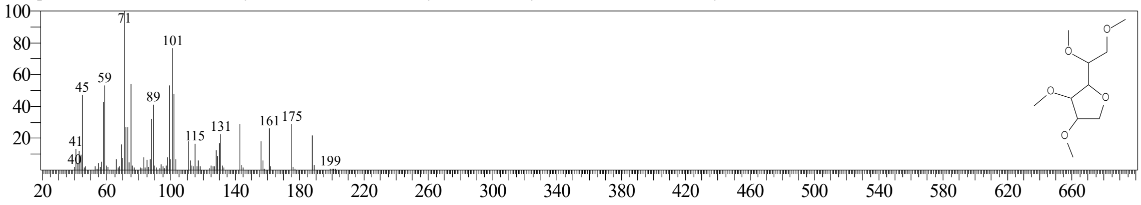

Hit#:2 Entry:46036 Library:NIST11.lib
SI:43 Formula:C9H16O5 CAS:0-00-0 MolWeight:204 RetIndex:1262
CompName:Hexanedioic acid, 3-methoxy-, dimethyl ester $$ Dimethyl 3-methoxyhexanedioate # $$

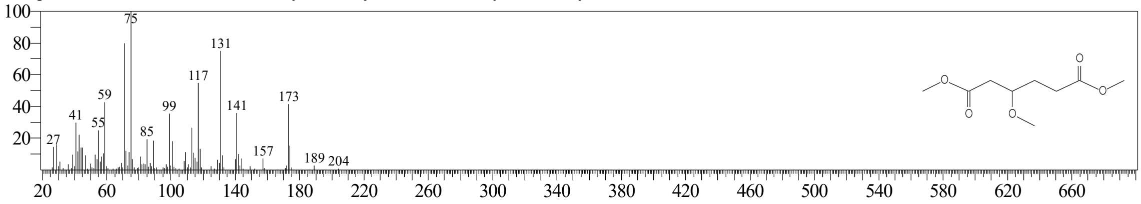

Hit#:3 Entry:20210 Library:NIST11.lib
SI:42 Formula:C8H16O3 CAS:2305-25-1 MolWeight:160 RetIndex:1146
CompName:Hexanoic acid, 3-hydroxy-, ethyl ester $$ Ethyl 3-hydroxyhexanoate $$ Ethyl hydroxy-3-hexanoate $$

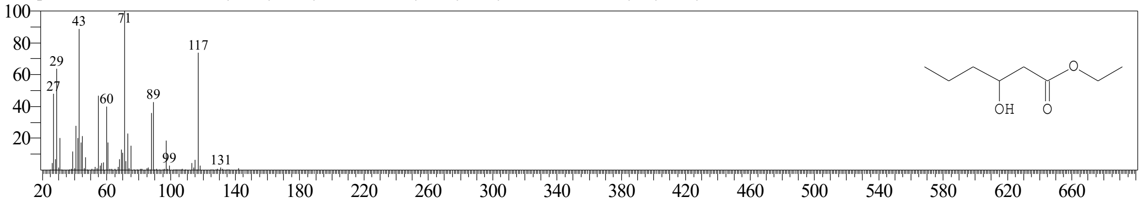

Hit#:4 Entry:188074 Library:NIST11.lib
SI:41 Formula:C27H44O3 CAS:29261-12-9 MolWeight:416 RetIndex:3204
CompName:9,10-Secocholesta-5,7,10(19)-triene-3,25,26-triol, (3.beta.,5Z,7E)- $$ 9,10-Secocholesta-5,7,10(19)-triene-3.beta.,25,26-triol $$ 25.Xi.,26-Dihydrc

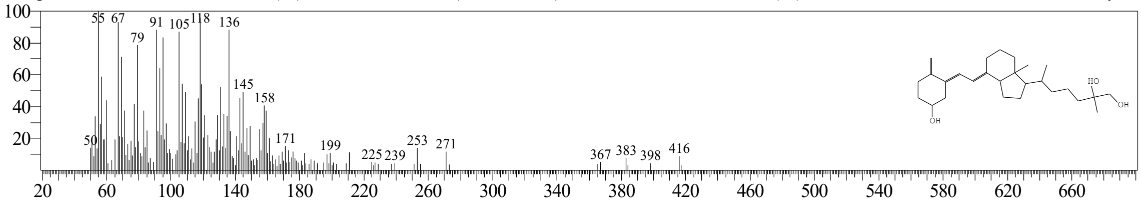

Hit#:5 Entry:13688 Library:NIST11.lib
SI:40 Formula:C7H14O3 CAS:54074-85-0 MolWeight:146 RetIndex:1047
CompName:Pentanoic acid, 3-hydroxy-, ethyl ester $$ Ethyl 3-hydroxypentanoate # $$

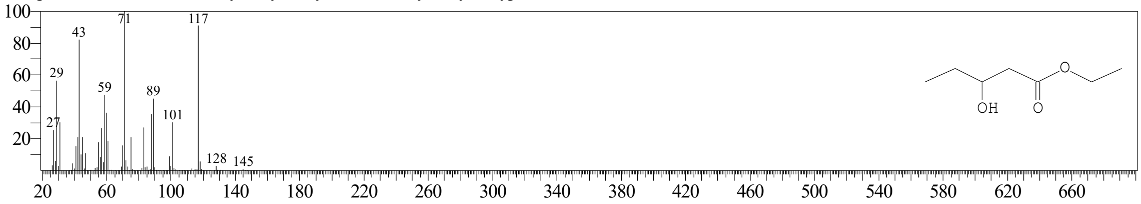

<< Target >>
Line#:15 R.Time:14.900(Scan#:2281) MassPeaks:370
RawMode:Averaged 14.895-14.905(2280-2282) BasePeak:151.10(1089)
BG Mode:Calc. from Peak Group 1 - Event 1 Scan

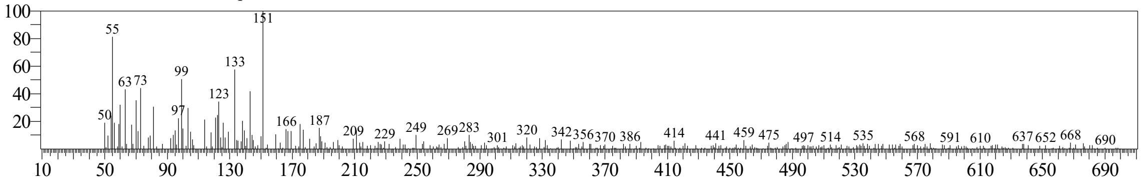

Hit#:1 Entry:73375 Library:NIST11.lib
SI:49 Formula:C12H18O5 CAS:0-00-0 MolWeight:242 RetIndex:1745
CompName:1,3-Cyclohexanediacetic acid, 2-oxo-, dimethyl ester

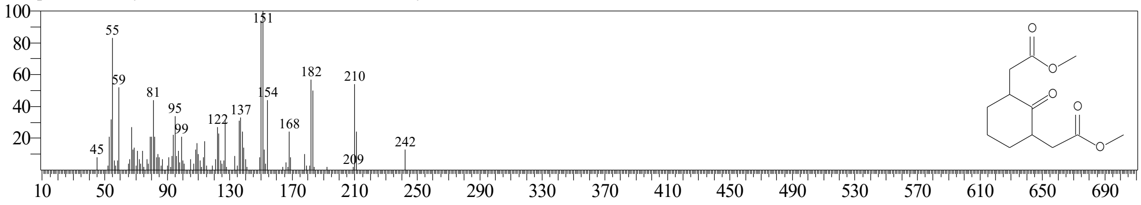

Hit#:2 Entry:57347 Library:NIST11.lib
SI:48 Formula:C12H12O4 CAS:0-00-0 MolWeight:220 RetIndex:1424
CompName:Fumaric acid, di(but-3-yn-2-yl) ester

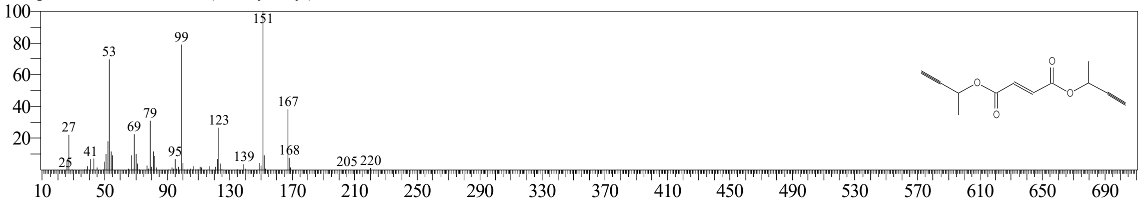

Hit#:3 Entry:18416 Library:NIST11.lib
SI:48 Formula:C9H16O2 CAS:4124-88-3 MolWeight:156 RetIndex:1280
CompName:3-Nonenoic acid $$ (3E)-3-Nonenoic acid # $$

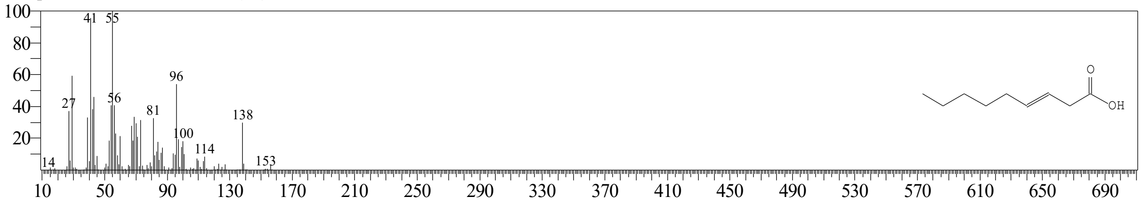

Hit#:4 Entry:12088 Library:NIST11.lib
SI:47 Formula:C8H14O2 CAS:0-00-0 MolWeight:142 RetIndex:1198
CompName:3-Methylcyclohexane-1-carboxylic acid $$ 3-Methylcyclohexanecarboxylic acid # $$

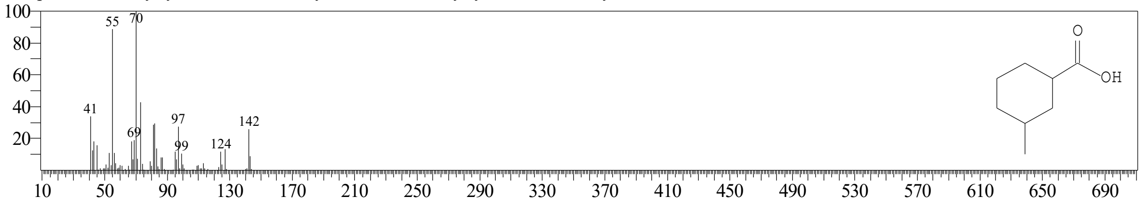

Hit#:5 Entry:175032 Library:NIST11.lib
SI:47 Formula:C21H36O6 CAS:63557-55-1 MolWeight:384 RetIndex:2963
CompName:Prost-13-en-1-oic acid, 9,11,15-trihydroxy-6-oxo-, methyl ester, (9.alpha.,11.alpha.,13E,15S)- $$ Methyl (13E)-9,11,15-trihydroxy-6-oxoprost-13-e

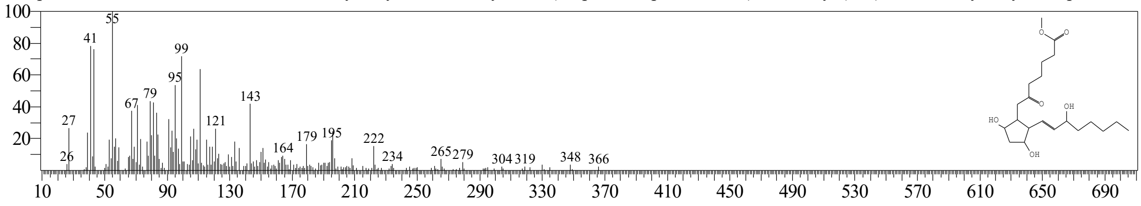

<< Target >>
Line#:16 R.Time:14.970(Scan#:2295) MassPeaks:337
RawMode:Averaged 14.965-14.975(2294-2296) BasePeak:73.05(741)
BG Mode:Calc. from Peak Group 1 - Event 1 Scan

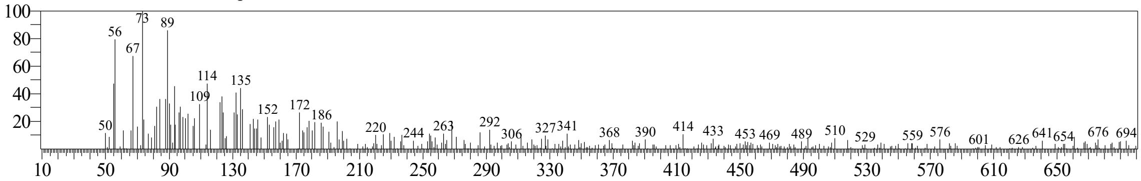

Hit#:1 Entry:75548 Library:NIST11.lib
SI:47 Formula:C11H19NOS2 CAS:116454-72-9 MolWeight:245 RetIndex:1918
CompName:Pyrrolidin-2-one, 5-[3-ethylenedithio-1-pentyl]- $$ 5-[2-(2-Ethyl-1,3-dithiolan-2-yl)ethyl]-2-pyrrolidinone # $$

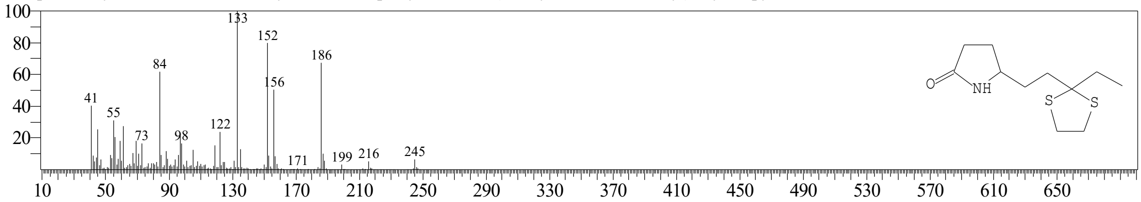

Hit#:2 Entry:42766 Library:NIST11.lib
SI:45 Formula:C12H22O2 CAS:76649-16-6 MolWeight:198 RetIndex:1389
CompName:Ethyl trans-4-decenoate $$ 4-Decenoic acid, ethyl ester, (E)- $$ Ethyl (4E)-4-decenoate $$ Ethyl 4E-decenoate $$ Ethyl (E)-4-decenoate $$ Ethyl tr

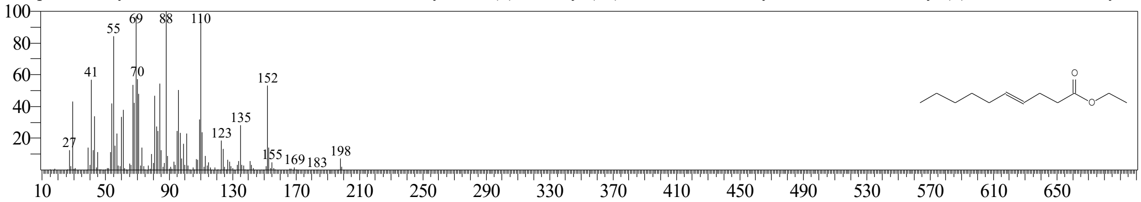

Hit#:3 Entry:52287 Library:NIST11.lib
SI:45 Formula:C13H24O2 CAS:692-86-4 MolWeight:212 RetIndex:1471
CompName:10-Undecenoic acid, ethyl ester $$ Ethyl undecenoate $$ Ethyl undecylenate $$ Ethyl 10-undecenoate $$ Undecenoic acid, ethyl ester $$ Ethyl 10-l

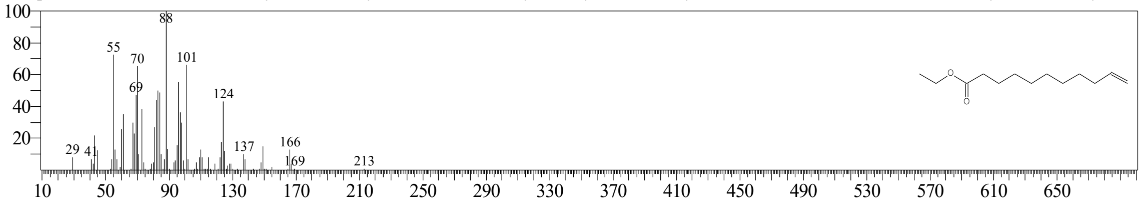

Hit#:4 Entry:175124 Library:NIST11.lib
SI:45 Formula:C22H44O3Si CAS:0-00-0 MolWeight:384 RetIndex:2305
CompName:13-Trimethylsilyloxy-9-octadecenoic acid, methyl ester $$ Methyl (9Z)-11-[(trimethylsilyl)oxy]-9-octadecenoate # $$

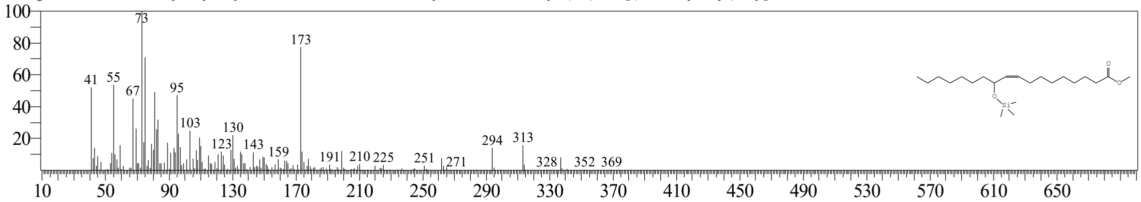

Hit#:5 Entry:195610 Library:NIST11.lib
SI:45 Formula:C28H44O4 CAS:56052-99-4 MolWeight:444 RetIndex:3145
CompName:Ergost-25-ene-3,6-dione, 5,12-dihydroxy-, (5.alpha.,12.beta.)- $$ 5,12-Dihydroxyergost-25(27)-ene-3,6-dione # $$

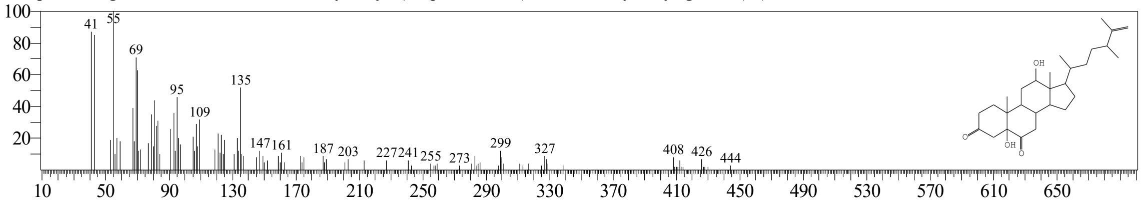

<< Target >>
Line#:17 R.Time:15.490(Scan#:2399) MassPeaks:394
RawMode:Averaged 15.485-15.495(2398-2400) BasePeak:73.10(872)
BG Mode:Calc. from Peak Group 1 - Event 1 Scan

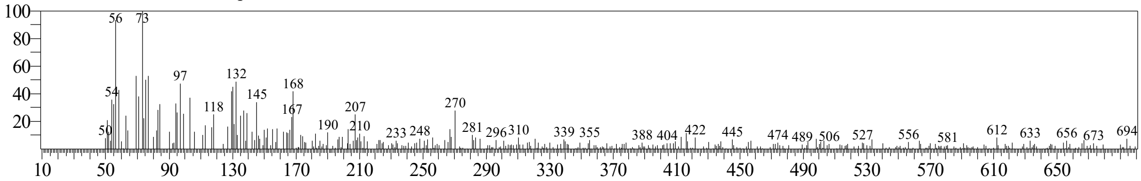

Hit#:1 Entry:135137 Library:NIST11.lib
SI:55 Formula:C13H27BrO2Si CAS:34176-84-6 MolWeight:322 RetIndex:1687
CompName:Decanoic acid, 10-bromo-, trimethylsilyl ester $$ Trimethylsilyl 10-bromodecanoate # $$

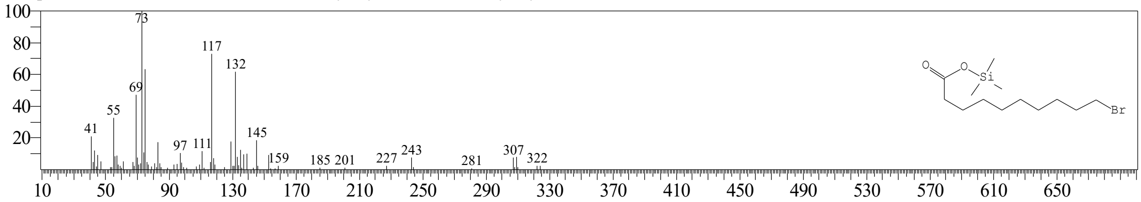

Hit#:2 Entry:145681 Library:NIST11.lib
SI:55 Formula:C14H29BrO2Si CAS:34176-85-7 MolWeight:336 RetIndex:1786
CompName:Undecanoic acid, 11-bromo-, trimethylsilyl ester $$ Trimethylsilyl 11-bromoundecanoate # $$

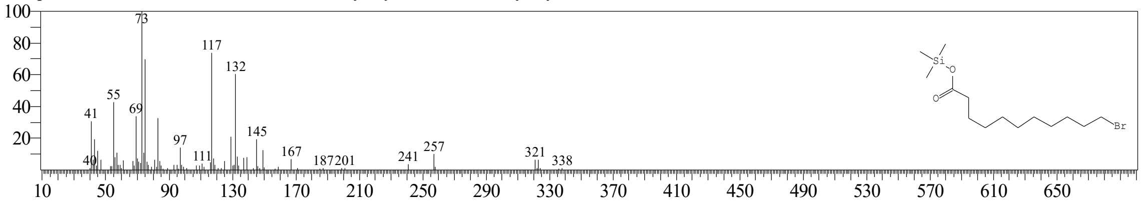

Hit#:3 Entry:88574 Library:NIST11.lib
SI:54 Formula:C13H27FO2Si CAS:26305-85-1 MolWeight:262 RetIndex:1364
CompName:Decanoic acid, 10-fluoro-, trimethylsilyl ester $$ Trimethylsilyl 10-fluorodecanoate # $$

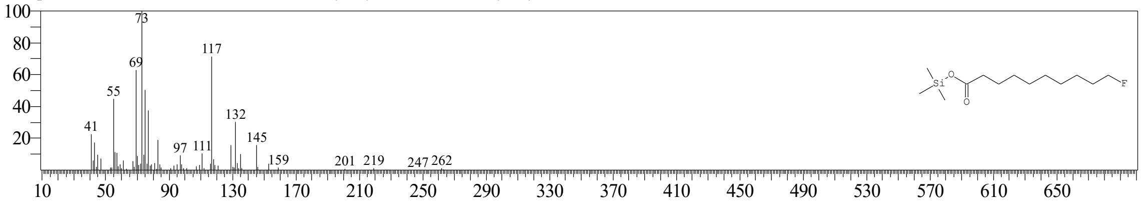

Hit#:4 Entry:99576 Library:NIST11.lib
SI:54 Formula:C14H29FO2Si CAS:26305-97-5 MolWeight:276 RetIndex:1463
CompName:Undecanoic acid, 11-fluoro-, trimethylsilyl ester $$ Trimethylsilyl 11-fluoroundecanoate # $$

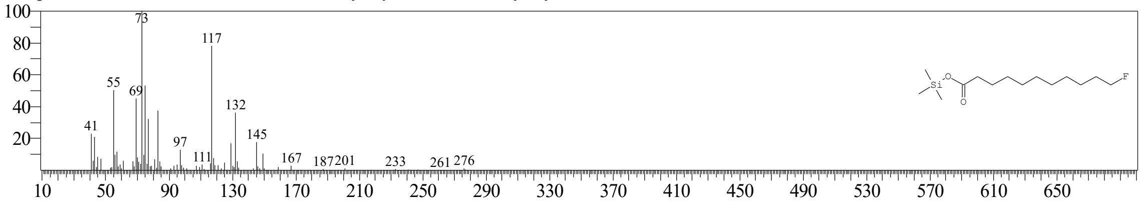

Hit#:5 Entry:194131 Library:NIST11.lib
SI:53 Formula:C27H54O2Si CAS:0-00-0 MolWeight:438 RetIndex:2790
CompName:cis-15-Tetracosenoic acid, trimethylsilyl ester

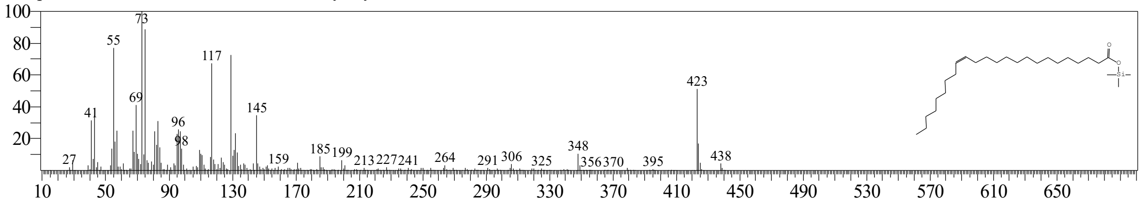

<< Target >>
Line#:18 R.Time:15.800(Scan#:2461) MassPeaks:337
RawMode:Averaged 15.795-15.805(2460-2462) BasePeak:55.10(1180)
BG Mode:Calc. from Peak Group 1 - Event 1 Scan

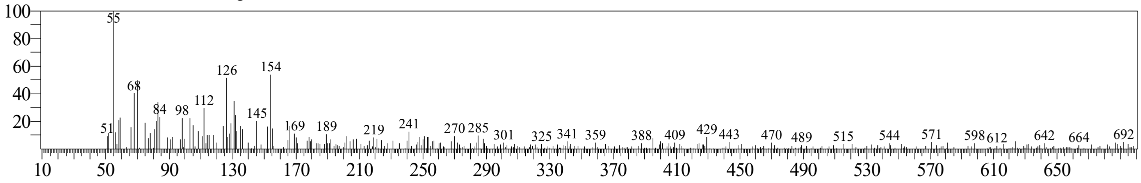

Hit#:1 Entry:17496 Library:NIST11.lib
SI:54 Formula:C10H18O CAS:16519-68-9 MolWeight:154 RetIndex:1212
CompName:Cyclohexanone, 2,6-diethyl- $$ 2,6-Diethylcyclohexanone $$

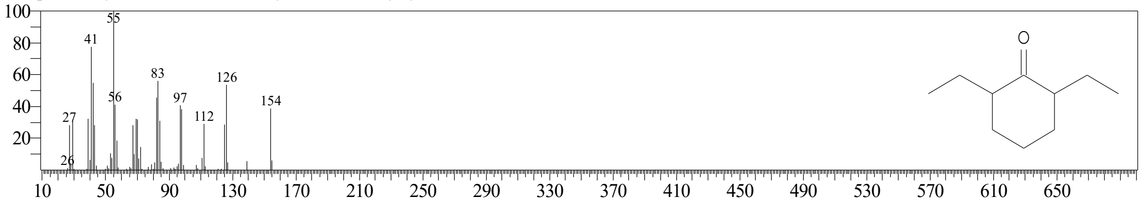

Hit#:2 Entry:17052 Library:NIST11.lib
SI:53 Formula:C7H10N2O2 CAS:19179-12-5 MolWeight:154 RetIndex:1404
CompName:Pyrrolo[1,2-a]pyrazine-1,4-dione, hexahydro- $$ Hexahydropyrrolo[1,2-a]pyrazine-1,4-dione $$ Pyrrolidino[1,2-a]piperazine-3,6-dione $$

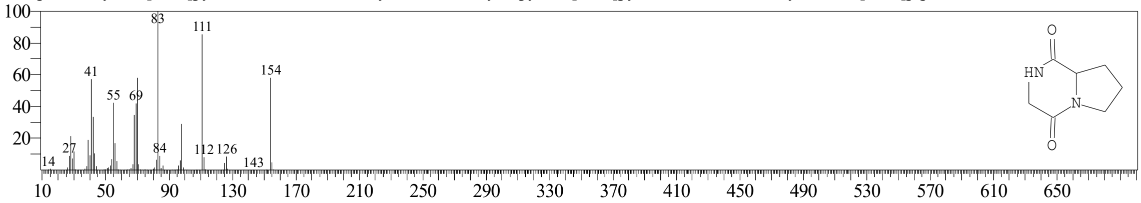

Hit#:3 Entry:17150 Library:NIST11.lib
SI:52 Formula:C8H10O3 CAS:75568-74-0 MolWeight:154 RetIndex:1153
CompName:2,6-Dioxa-tricyclo[3.3.2.0(3,7)]decan-9-one

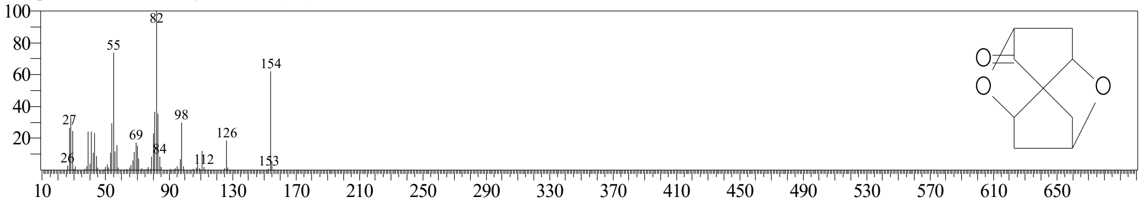

Hit#:4 Entry:35307 Library:NIST11.lib
SI:51 Formula:C12H26O CAS:7289-53-4 MolWeight:186 RetIndex:1290
CompName:Methyl undecyl ether $$ 1-Methoxyundecane # $$

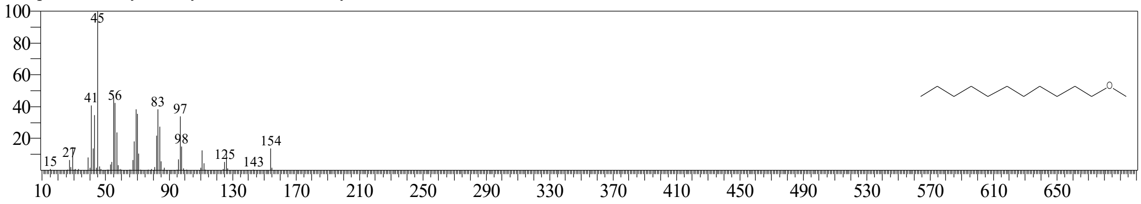

Hit#:5 Entry:25228 Library:NIST11.lib
SI:51 Formula:C10H19NO CAS:0-00-0 MolWeight:169 RetIndex:1202
CompName:N-(1,1-Dimethyl-3-oxobutyl)-2-methylazetidine $$ 4-Methyl-4-(2-methyl-1-azetidinyl)-2-pentanone # $$

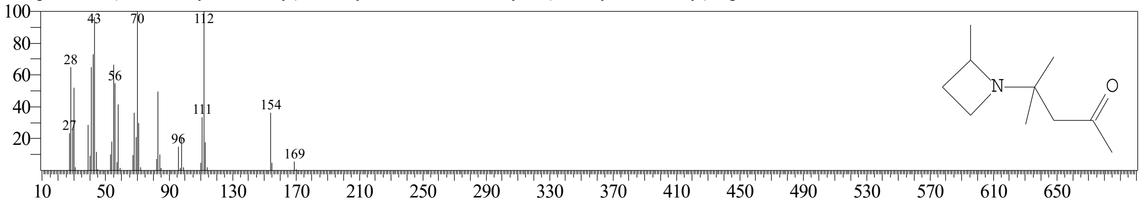

Qualitative Analysis Report

<< Target >>
Line#:19 R.Time:15.885(Scan#:2478) MassPeaks:372
RawMode:Averaged 15.880-15.890(2477-2479) BasePeak:111.10(777)
BG Mode:Calc. from Peak Group 1 - Event 1 Scan

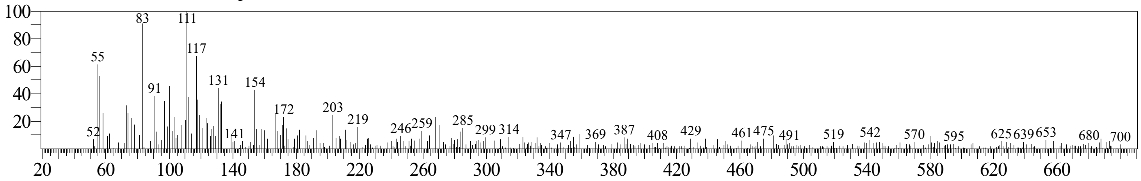

Hit#:1 Entry:76206 Library:NIST11.lib
SI:47 Formula:C11H18O6 CAS:50326-91-5 MolWeight:246 RetIndex:1867
CompName:2H-Furo[3,2-b]pyran-2-one, hexahydro-3,4(or 3,8)-dihydroxy-8(or 4)-methoxy-6,7,8-trimethyl-

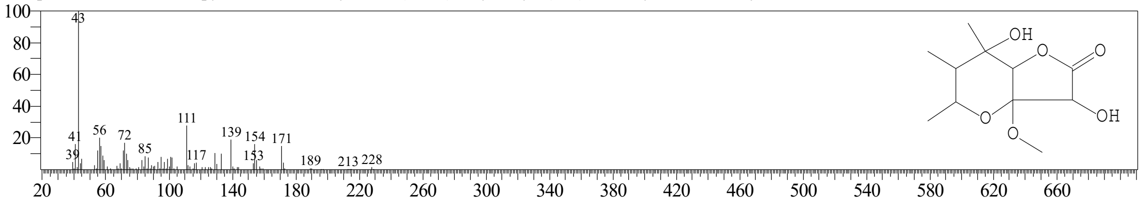

Hit#:2 Entry:148807 Library:NIST11.lib
SI:46 Formula:C20H36O4 CAS:0-00-0 MolWeight:340 RetIndex:2288
CompName:Fumaric acid, 2-butyl dodecyl ester

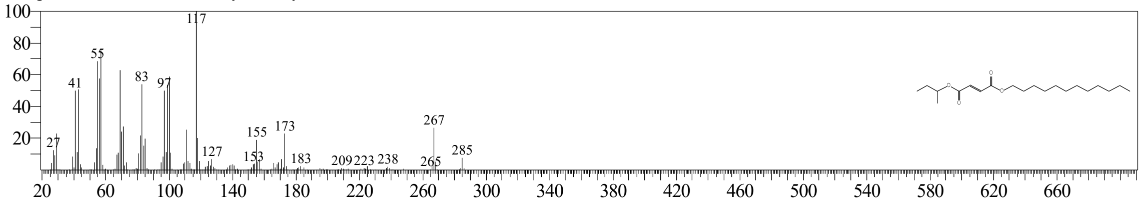

Hit#:3 Entry:64429 Library:NIST11.lib
SI:46 Formula:C6H5Cl3O3 CAS:0-00-0 MolWeight:230 RetIndex:1246
CompName:Carbonic acid, propargyl 2,2,2-trichloroethyl ester

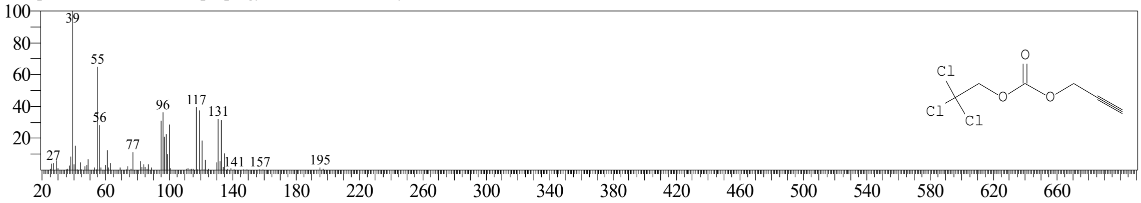

Hit#:4 Entry:127852 Library:NIST11.lib
SI:45 Formula:C18H32O4 CAS:0-00-0 MolWeight:312 RetIndex:2089
CompName:Fumaric acid, 2-butyl decyl ester

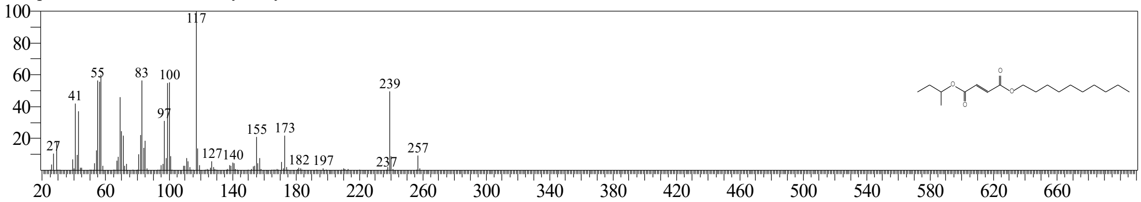

Hit#:5 Entry:138455 Library:NIST11.lib
SI:45 Formula:C19H34O4 CAS:0-00-0 MolWeight:326 RetIndex:2188
CompName:Fumaric acid, 2-butyl undecyl ester

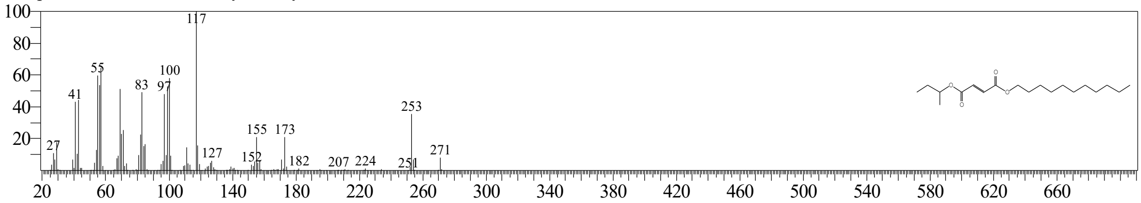

<< Target >>
Line#:20 R.Time:16.275(Scan#:2556) MassPeaks:341
RawMode:Averaged 16.270-16.280(2555-2557) BasePeak:70.05(5525)
BG Mode:Calc. from Peak Group 1 - Event 1 Scan

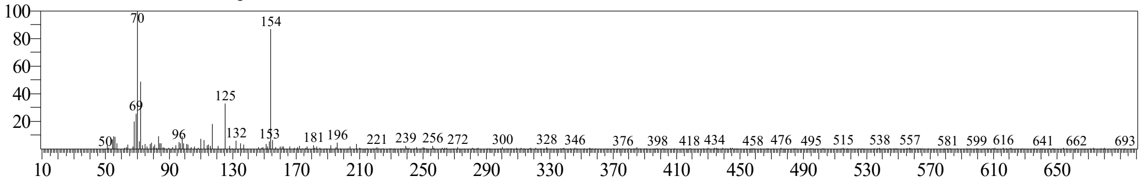

Hit#:1 Entry:50619 Library:NIST11.lib
SI:73 Formula:C11H18N2O2 CAS:5654-86-4 MolWeight:210 RetIndex:1699
CompName:Pyrrolo[1,2-a]pyrazine-1,4-dione, hexahydro-3-(2-methylpropyl)- $$ 3-Isobutylhexahydropyrrolo[1,2-a]pyrazine-1,4-dione $$ Cyclo(leucyloprolyl)

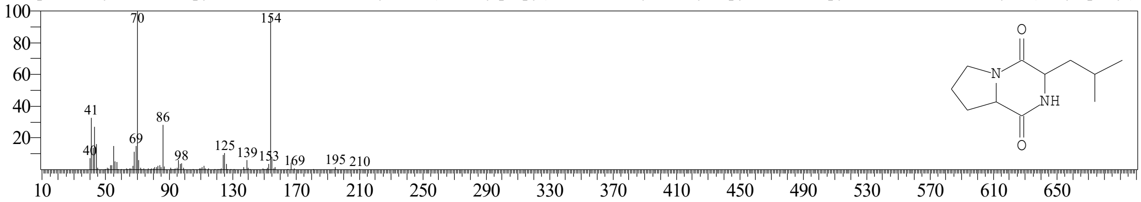

Hit#:2 Entry:197003 Library:NIST11.lib
SI:68 Formula:C28H53NO3 CAS:0-00-0 MolWeight:451 RetIndex:3264
CompName:L-Proline, N-valeryl-, octadecyl ester

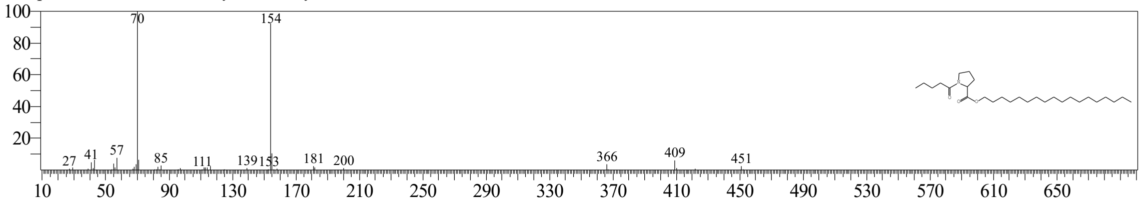

Hit#:3 Entry:193770 Library:NIST11.lib
SI:68 Formula:C27H51NO3 CAS:0-00-0 MolWeight:437 RetIndex:3164
CompName:L-Proline, N-valeryl-, heptadecyl ester

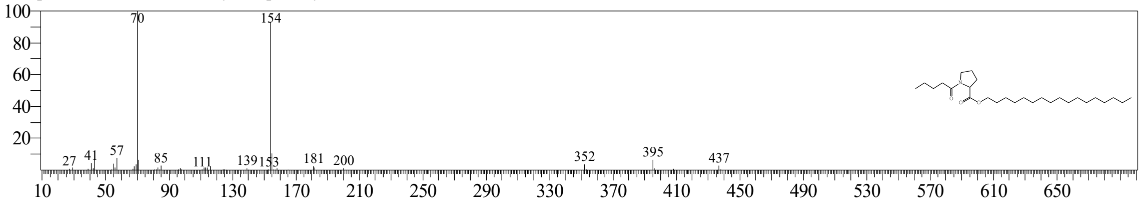

Hit#:4 Entry:185514 Library:NIST11.lib
SI:67 Formula:C25H47NO3 CAS:0-00-0 MolWeight:409 RetIndex:2966
CompName:L-Proline, N-valeryl-, pentadecyl ester

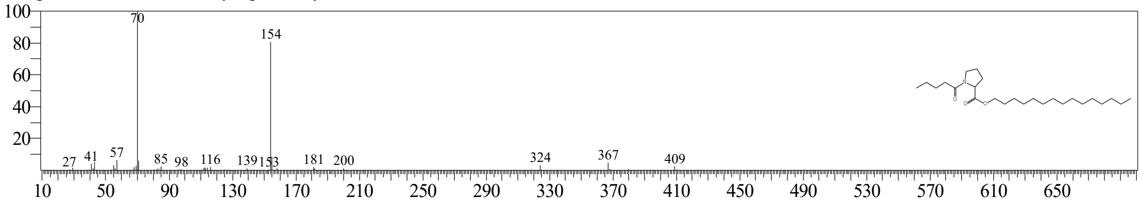

Hit#:5 Entry:17187 Library:NIST11.lib
SI:67 Formula:C8H14N2O CAS:76284-12-3 MolWeight:154 RetIndex:1317
CompName:5-Pyrrolidino-2-pyrrolidone

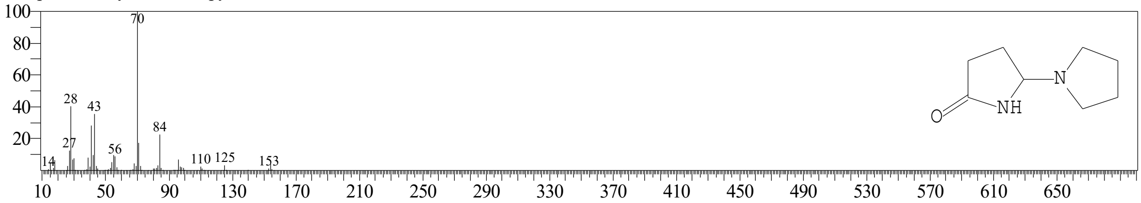

<< Target >>
Line#:21 R.Time:16.695(Scan#:2640) MassPeaks:367
RawMode:Averaged 16.690-16.700(2639-2641) BasePeak:154.15(753)
BG Mode:Calc. from Peak Group 1 - Event 1 Scan

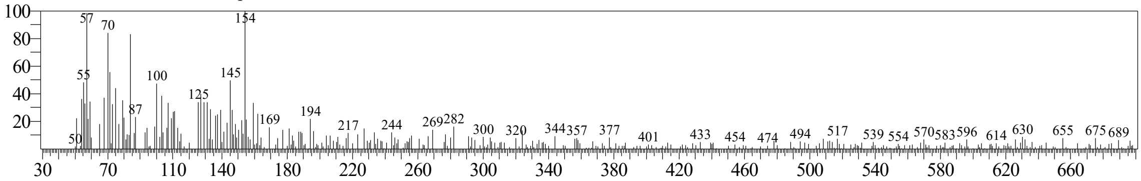

Hit#:1 Entry:20037 Library:NIST11.lib
SI:50 Formula:C7H12O4 CAS:16503-06-3 MolWeight:160 RetIndex:1221
CompName:Pentanoic acid, 2-(methoxymethyl)-4-oxo- $$ 2-(Methoxymethyl)-4-oxopentanoic acid # $$

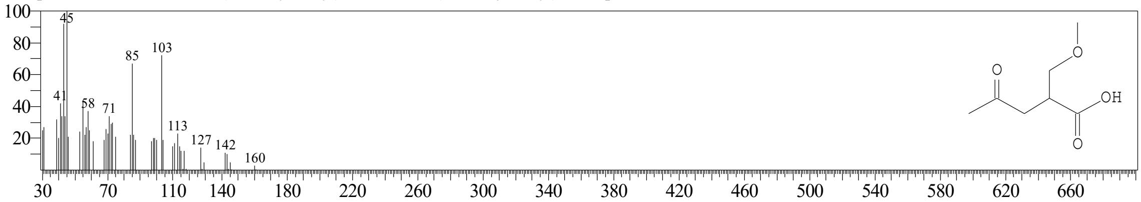

Hit#:2 Entry:17368 Library:NIST11.lib
SI:50 Formula:C9H14O2 CAS:42117-32-8 MolWeight:154 RetIndex:1173
CompName:2-Methoxy-4,4-dimethyl-2-cyclohexen-1-one

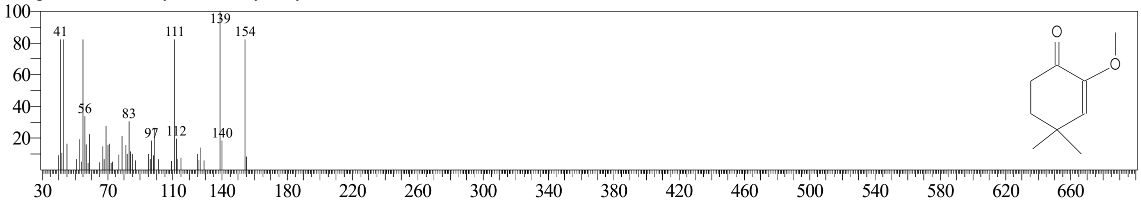

Hit#:3 Entry:50868 Library:NIST11.lib
SI:50 Formula:C13H22O2 CAS:0-00-0 MolWeight:210 RetIndex:1440
CompName:1-Ethynyl-3,cis(1,1-dimethylethyl)-4,trans-methoxycyclohexan-1-ol $$ 3-tert-Butyl-1-ethynyl-4-methoxycyclohexanol # $$

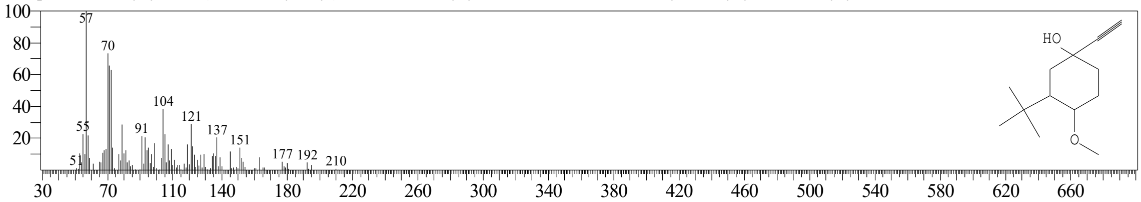

Hit#:4 Entry:50869 Library:NIST11.lib
SI:49 Formula:C13H22O2 CAS:0-00-0 MolWeight:210 RetIndex:1440
CompName:1-Ethynyl-3,trans(1,1-dimethylethyl)-4,cis-methoxycyclohexan-1-ol $$ 3-tert-Butyl-1-ethynyl-4-methoxycyclohexanol # $$

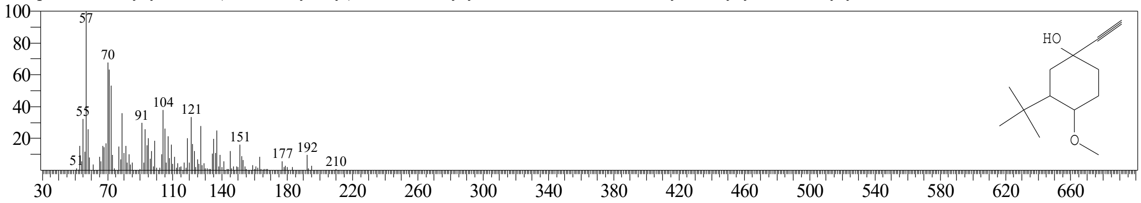

Hit#:5 Entry:43020 Library:NIST11.lib
SI:49 Formula:C7H9N3O2S CAS:115154-37-5 MolWeight:199 RetIndex:1909
CompName:4-Thiazolebutanamide, 2-amino-.gamma.-oxo- $$ 4-(2-Amino-1,3-thiazol-4-yl)-4-oxobutanamide # $$

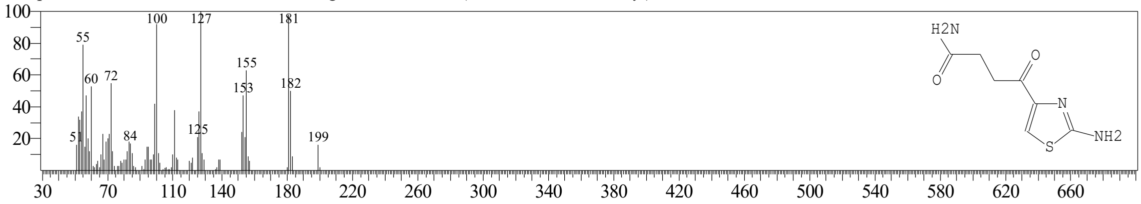

<< Target >>
Line#:22 R.Time:18.010(Scan#:2903) MassPeaks:330
RawMode:Averaged 18.005-18.015(2902-2904) BasePeak:154.10(1274)
BG Mode:Calc. from Peak Group 1 - Event 1 Scan

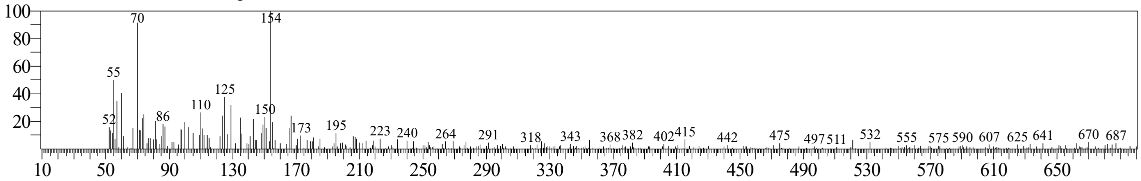

Hit#:1 Entry:52550 Library:NIST11.lib
SI:55 Formula:C7H11N5O3 CAS:347355-79-7 MolWeight:213 RetIndex:2149
CompName:4-Amino-furazan-3-carboxylic acid (2-acetylamino-ethyl)-amide $$ N-[2-(Acetylamino)ethyl]-4-amino-1,2,5-oxadiazole-3-carboxamide # $$

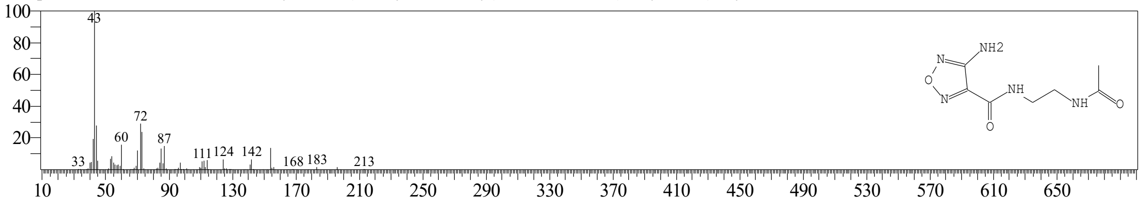

Hit#:2 Entry:53448 Library:NIST11.lib
SI:54 Formula:C12H22O3 CAS:34208-02-1 MolWeight:214 RetIndex:1506
CompName:Nonanoic acid, 9-oxo-, 1-methylethyl ester $$ Isopropyl 9-oxononanoate # $$

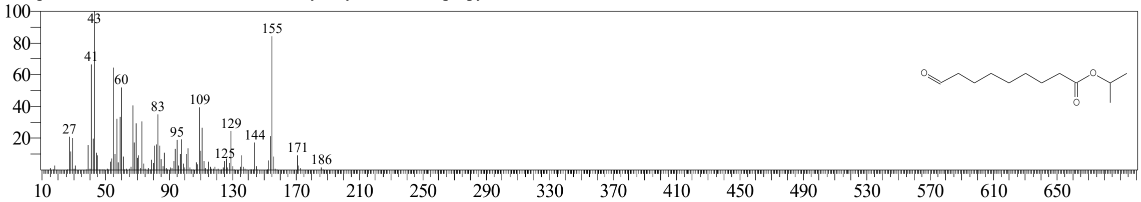

Hit#:3 Entry:73875 Library:NIST11.lib
SI:53 Formula:C15H30O2 CAS:0-00-0 MolWeight:242 RetIndex:1615
CompName:(-)-1-Methylbutyl decanoate $$ 1-Methylbutyl decanoate # $$

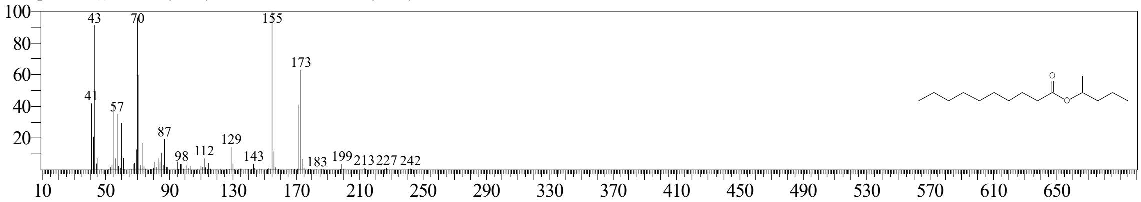

Hit#:4 Entry:73850 Library:NIST11.lib
SI:53 Formula:C15H30O2 CAS:0-00-0 MolWeight:242 RetIndex:1615
CompName:2-Pentanol decanoate $$ 1-Methylbutyl decanoate # $$

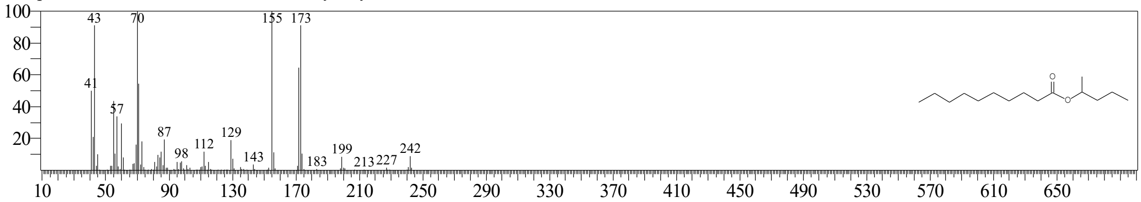

Hit#:5 Entry:16949 Library:NIST11.lib
SI:53 Formula:C5H6N4O2 CAS:147194-49-8 MolWeight:154 RetIndex:1470
CompName:3-Furazancarboxamide, 4-(1-aziridinyl)- $$ 4-(1-Aziridinyl)-1,2,5-oxadiazole-3-carboxamide # $$

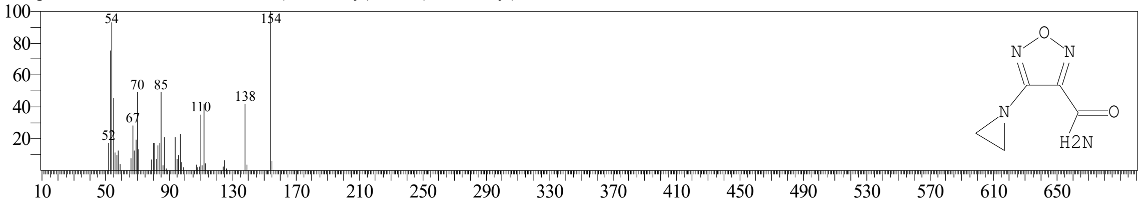

Qualitative Analysis Report

<< Target >>
Line#:23 R.Time:19.410(Scan#:3183) MassPeaks:386
RawMode:Averaged 19.405-19.415(3182-3184) BasePeak:68.10(424)
BG Mode:Calc. from Peak Group 1 - Event 1 Scan

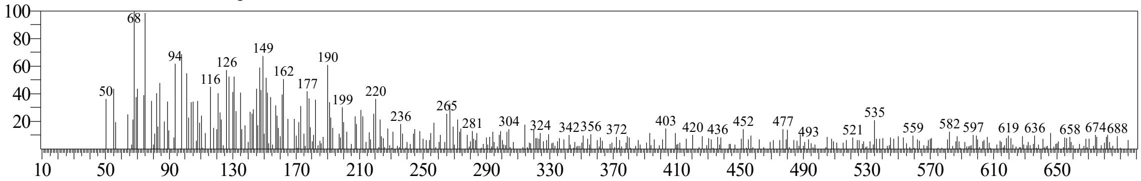

Hit#:1 Entry:70739 Library:NIST11.lib
SI:40 Formula:C15H26O2 CAS:88588-48-1 MolWeight:238 RetIndex:1786
CompName:Cedran-diol, 8S,13- $$ Cedrane-8,13-diol # $$

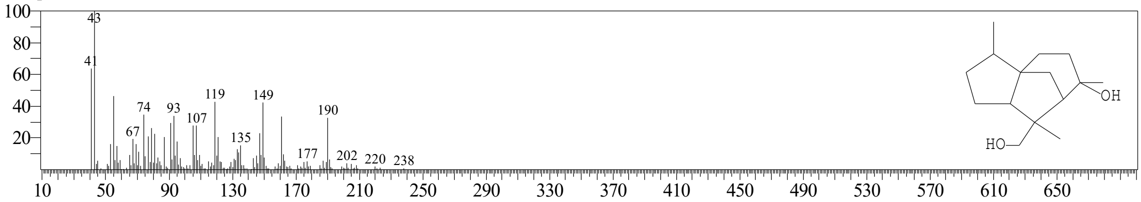

Hit#:2 Entry:134576 Library:NIST11.lib
SI:39 Formula:C15H16ClN3O3 CAS:0-00-0 MolWeight:321 RetIndex:2676
CompName:1H-Pyrazole-1-acetamide, N-(1,3-benzodioxol-5-ylmethyl)-4-chloro-3,5-dimethyl-

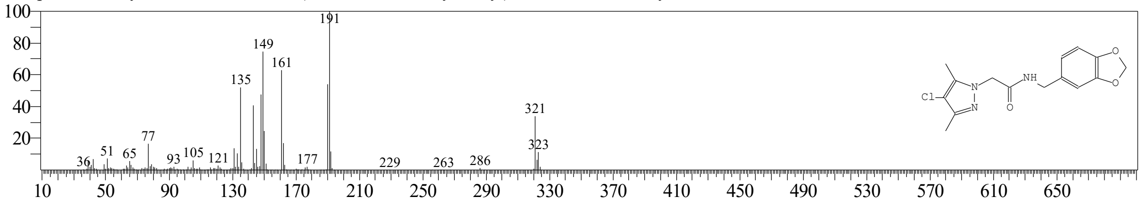

Hit#:3 Entry:153704 Library:NIST11.lib
SI:39 Formula:C21H37NOSi CAS:0-00-0 MolWeight:347 RetIndex:2220
CompName:(-)-Isolongifolol, (3-cyanopropyl)dimethylsilyl ether

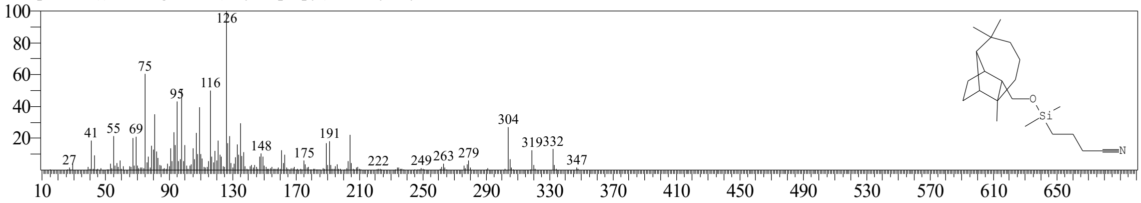

Hit#:4 Entry:102501 Library:NIST11.lib
SI:38 Formula:C15H24O3Si CAS:0-00-0 MolWeight:280 RetIndex:1756
CompName:(2-Methoxyphenyl)pentanoic acid, trimethylsilyl (ester) $$ Trimethylsilyl 5-(2-methoxyphenyl)pentanoate # $$

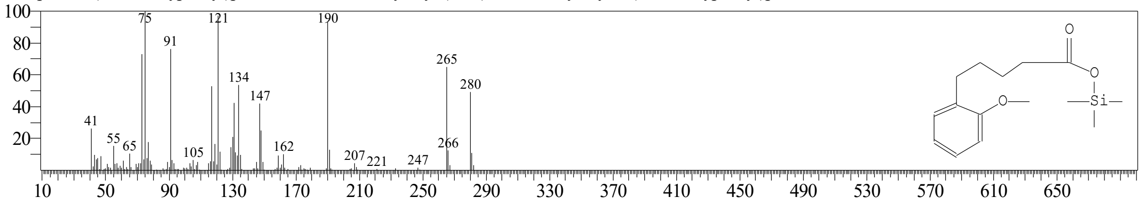

Hit#:5 Entry:102500 Library:NIST11.lib
SI:38 Formula:C15H24O3Si CAS:0-00-0 MolWeight:280 RetIndex:1756
CompName:(3-Methoxyphenyl)pentanoic acid, trimethylsilyl (ester) $$ Trimethylsilyl 5-(3-methoxyphenyl)pentanoate # $$

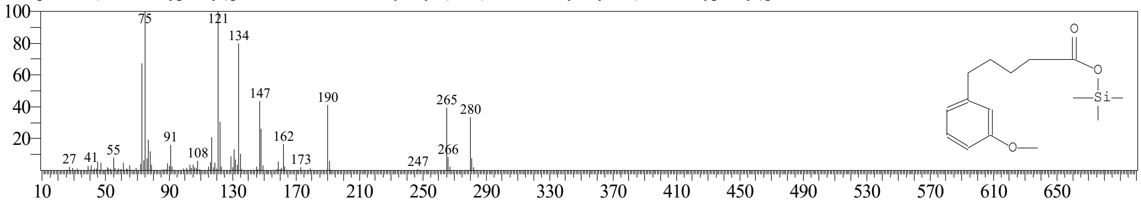

<< Target >>
Line#:24 R.Time:22.225(Scan#:3746) MassPeaks:395
RawMode:Averaged 22.220-22.230(3745-3747) BasePeak:56.95(470)
BG Mode:Calc. from Peak Group 1 - Event 1 Scan

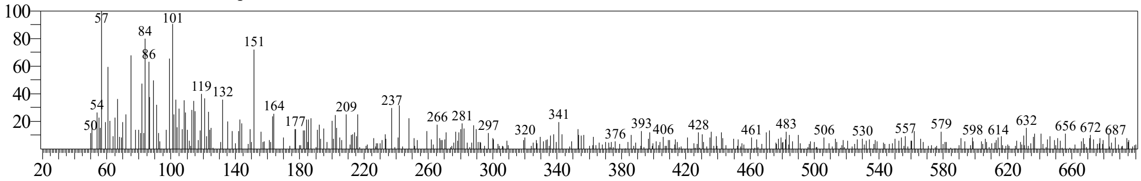

Hit#:1 Entry:89869 Library:NIST11.lib
SI:47 Formula:C12H24O6 CAS:109773-63-9 MolWeight:264 RetIndex:2196
CompName:3-(1,3-Dihydroxyisopropyl)-1,5,8,11-tetraoxacyclotridecane $$ 2-(1,4,7,10-Tetraoxacyclotridecan-12-yl)-1,3-propanediol # $$

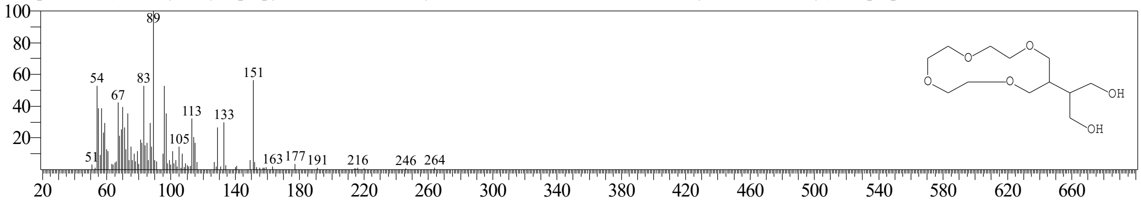

Hit#:2 Entry:124390 Library:NIST11.lib
SI:45 Formula:C14H28O7 CAS:109773-64-0 MolWeight:308 RetIndex:2545
CompName:3-(1,3-Dihydroxyisopropyl)-1,5,8,11,14-pentaoxacyclohexadecane $$ 2-(1,4,7,10,13-Pentaoxacyclohexadecan-15-yl)-1,3-propanediol # $$

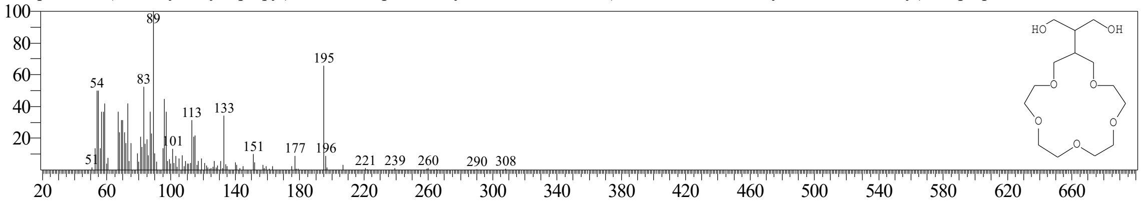

Hit#:3 Entry:189162 Library:NIST11.lib
SI:45 Formula:C21H40O8 CAS:120343-89-7 MolWeight:420 RetIndex:3145
CompName:3,3'-Isopropylidenebis(1,5,8,11-tetraoxacyclotridecane) $$ 12-[1-Methyl-1-(1,4,7,10-tetraoxacyclotridecan-12-yl)ethyl]-1,4,7,10-tetraoxacyclotride

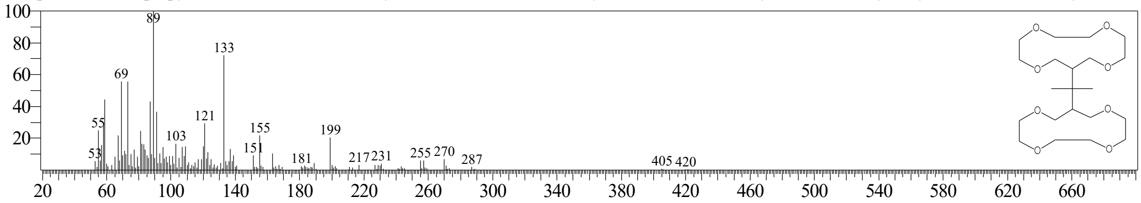

Hit#:4 Entry:88236 Library:NIST11.lib
SI:44 Formula:C10H14O8 CAS:79687-33-5 MolWeight:262 RetIndex:2453
CompName:1,4,7,10,13-Pentaoxacyclopentadecane-2,5,9-trione

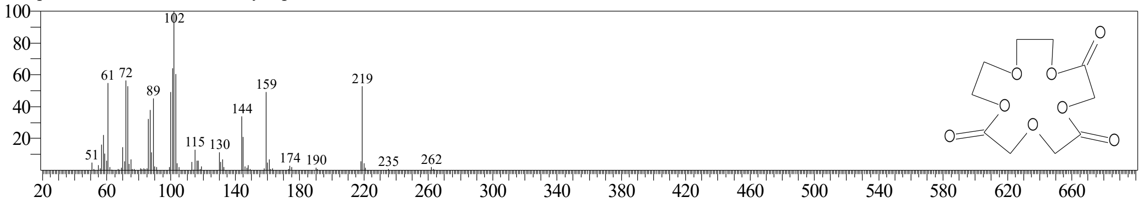

Hit#:5 Entry:43874 Library:NIST11.lib
SI:44 Formula:C11H24OSi CAS:0-00-0 MolWeight:200 RetIndex:1065
CompName:Allyloxydi(tert-butyl)silane

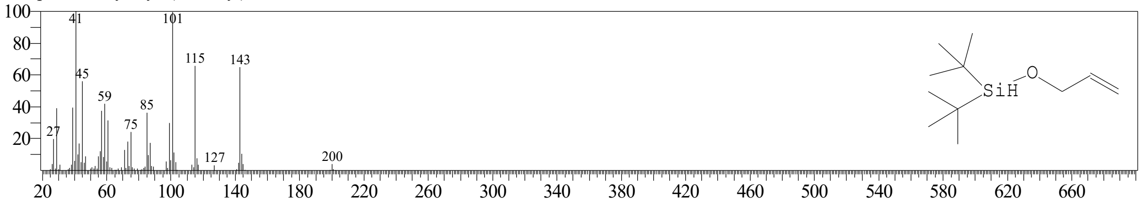

<< Target >>
Line#:25 R.Time:25.035(Scan#:4308) MassPeaks:348
RawMode:Averaged 25.030-25.040(4307-4309) BasePeak:91.05(478)
BG Mode:Calc. from Peak Group 1 - Event 1 Scan

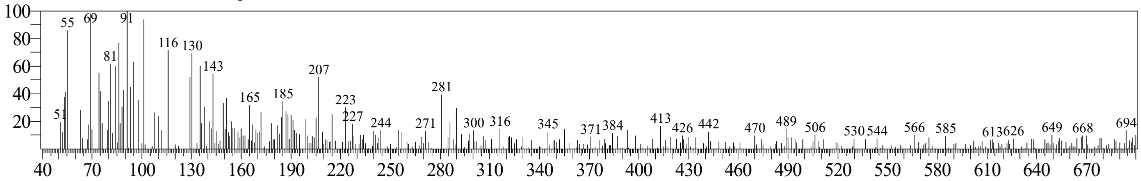

Hit#:1 Entry:170435 Library:NIST11.lib
SI:44 Formula:C25H42O2 CAS:56051-53-7 MolWeight:374 RetIndex:2528
CompName:Cyclopropanebutanoic acid, 2-[[2-[[2-[(2-pentylcyclopropyl)methyl]cyclopropyl]methyl]cyclopropyl]methyl]-, methyl ester $$ Methyl 4-(2-([2-((2-[

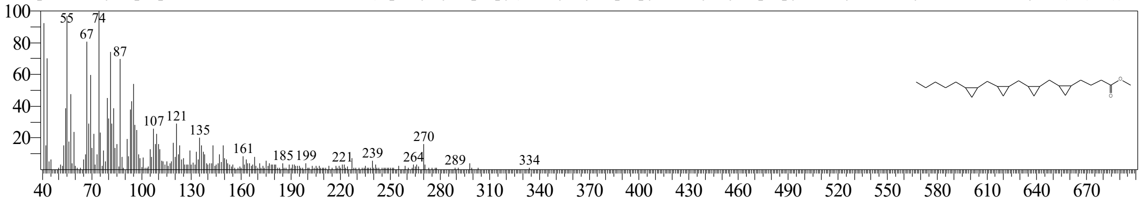

Hit#:2 Entry:182436 Library:NIST11.lib
SI:44 Formula:C22H44O4Si CAS:22032-78-6 MolWeight:400 RetIndex:2413
CompName:Octadecanoic acid, 9,10-epoxy-18-(trimethylsiloxy)-, methyl ester, cis- $$ Methyl 8-(3-(8-[(trimethylsilyl)oxy]octyl)-2-oxiranyl)octanoate # $$

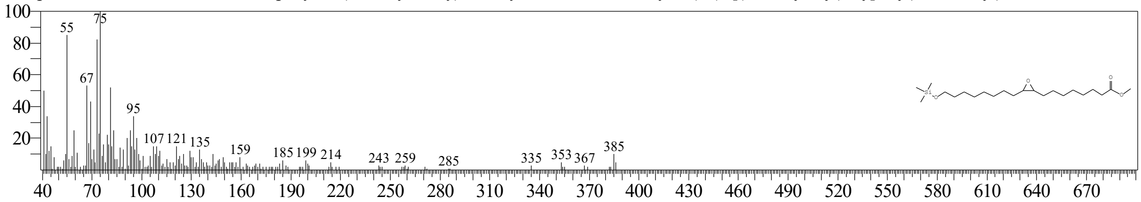

Hit#:3 Entry:52128 Library:NIST11.lib
SI:44 Formula:C12H20O3 CAS:30414-60-9 MolWeight:212 RetIndex:1443
CompName:Octanoic acid, 3-oxo-4-(2-propenyl)-, methyl ester $$ Octanoic acid, 4-allyl-3-oxo-, methyl ester $$ Methyl 4-butyl-3-oxo-6-heptenoate # $$

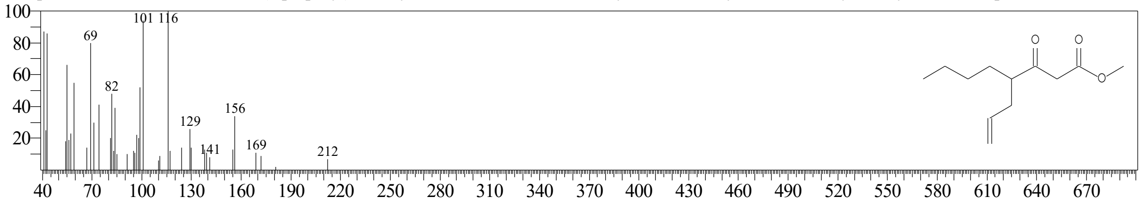

Hit#:4 Entry:107721 Library:NIST11.lib
SI:43 Formula:C17H34O3 CAS:0-00-0 MolWeight:286 RetIndex:2121
CompName:Methyl 16-hydroxy-hexadecanoate

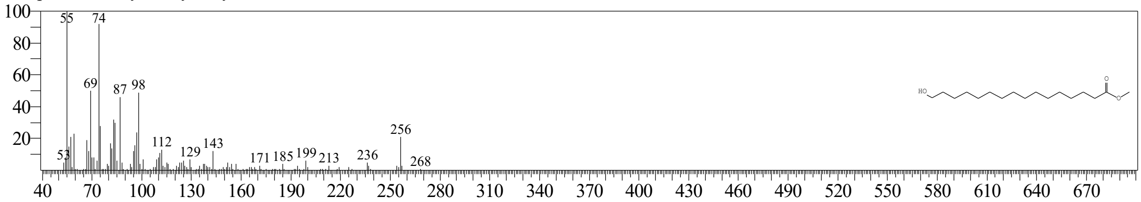

Hit#:5 Entry:27147 Library:NIST11.lib
SI:43 Formula:C10H20S CAS:76097-70-6 MolWeight:172 RetIndex:1281
CompName:trans-2-Methyl-4-n-butylthiane $$ 4-Butyl-2-methyltetrahydro-2H-thiopyran, trans $$

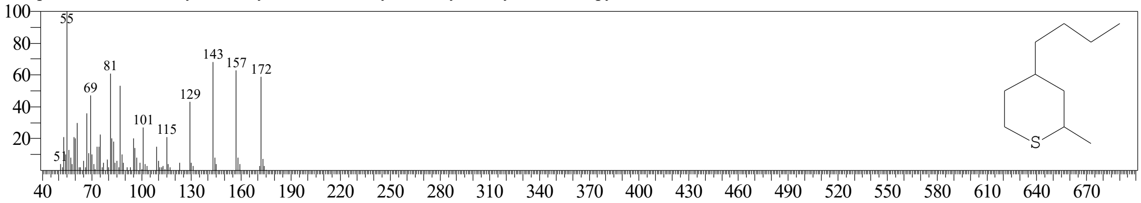

<< Target >>
Line#:26 R.Time:31.945(Scan#:5690) MassPeaks:355
RawMode:Averaged 31.940-31.950(5689-5691) BasePeak:67.10(510)
BG Mode:Calc. from Peak Group 1 - Event 1 Scan

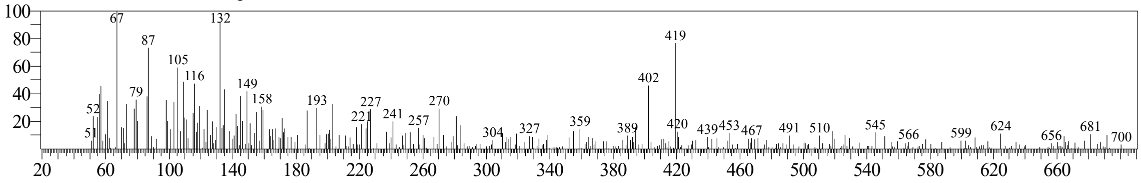

Hit#:1 Entry:99178 Library:NIST11.lib
SI:40 Formula:C10H16N2O5S CAS:74229-40-6 MolWeight:276 RetIndex:2416
CompName:1,7-Dioxa-10-thia-4,13-diazacyclopentadeca-5,9,12-trione $$ 1,7-Dioxa-10-thia-4,13-diazacyclopentadecane-5,9,12-trione # $$

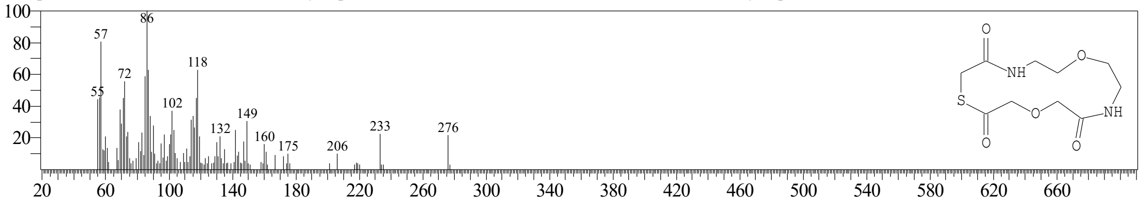

Hit#:2 Entry:155451 Library:NIST11.lib
SI:39 Formula:C17H34O5S CAS:0-00-0 MolWeight:350 RetIndex:2882
CompName:.alpha.,.beta.-D-Glucopyranoside, 1-deoxy-1-undecylthio- $$ Undecyl 1-thiohexopyranoside # $$

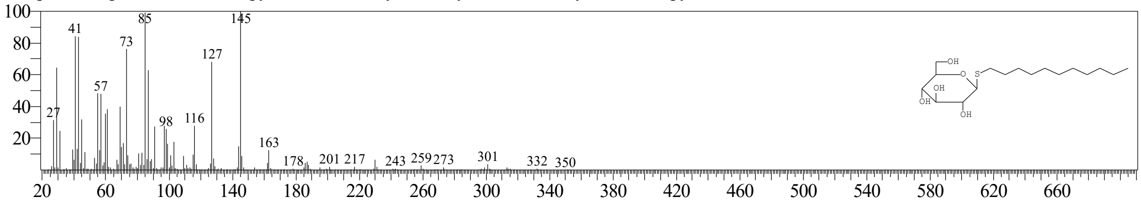

Hit#:3 Entry:45778 Library:NIST11.lib
SI:39 Formula:C14H21N CAS:77581-12-5 MolWeight:203 RetIndex:1752
CompName:2,11-Dimethyl-2,3,4,5,6,7-hexahydro-1H-2-benzazonine

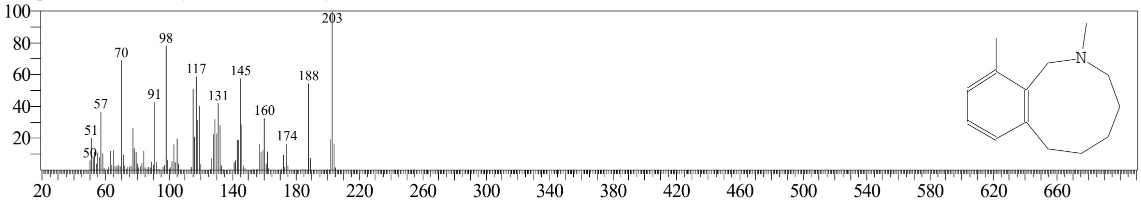

Hit#:4 Entry:114806 Library:NIST11.lib
SI:39 Formula:C13H28O5S CAS:0-00-0 MolWeight:296 RetIndex:2456
CompName:d-Galactitol, 1-thioheptyl-1-deoxy- $$ 1-S-Heptyl-1-thio-d-galactitol # $$

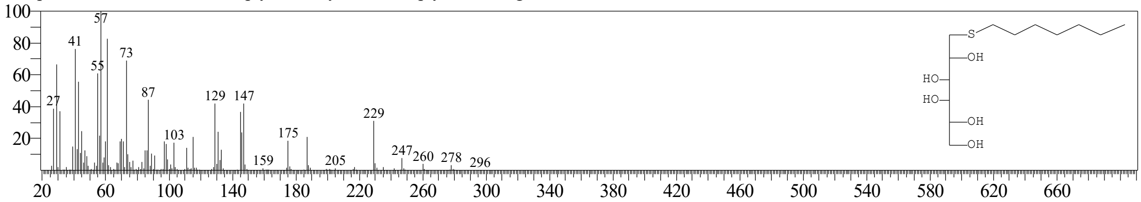

Hit#:5 Entry:106237 Library:NIST11.lib
SI:38 Formula:C19H24O2 CAS:4075-12-1 MolWeight:284 RetIndex:2103
CompName:Androsta-1,4,6-trien-3-one, 17-hydroxy-, (17.beta.)- $$ Androsta-1,4,6-trien-3-one, 17.beta.-hydroxy- $$ 1,4,6-Androstatrien-17.beta.-ol-3-one $$ 1

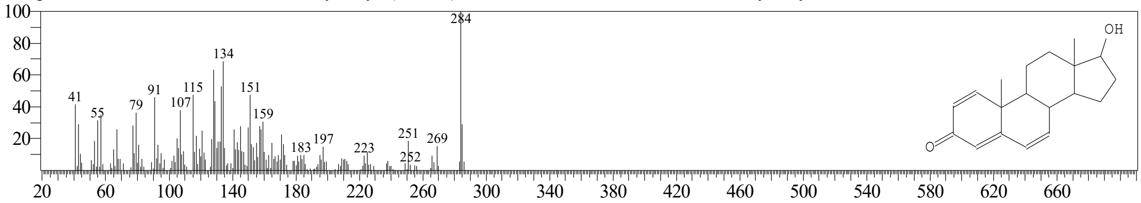

Supplement: Supplementary file 1 [file plants-12-00869-s001.zip › plants-2204717-supplementary.pdf]
